# Sequence-dependent synergistic effect of aumolertinib-pemetrexed combined therapy on EGFR-mutant non-small-cell lung carcinoma with pre-clinical and clinical evidence

**DOI:** 10.1186/s13046-022-02369-3

**Published:** 2022-05-03

**Authors:** Luyao Ao, Shencun Fang, Kexin Zhang, Yang Gao, Jiawen Cui, Wenjing Jia, Yunlong Shan, Jingwei Zhang, Guangji Wang, Jiali Liu, Fang Zhou

**Affiliations:** 1 Jiangsu Provincial Key Laboratory of Drug Metabolism and Pharmacokinetics, State Key Laboratory of Natural Medicines, China Pharmaceutical University, Tongjiaxiang #24, Nanjing, 210009 Jiangsu China; 2 Department of Respiratory Medicine, Nanjing Chest Hospital, The Affiliated Brain Hospital of Nanjing Medical University, 215 Guangzhou Road, Nanjing, 210029 Jiangsu China

**Keywords:** Aumolertinib, Pemetrexed, Sequential drug administration, Non-small cell lung cancer, Epidermal growth factor receptor, Synergistic effect

## Abstract

**Background:**

Inevitably developed resistance of the third-generation ﻿epidermal growth factor receptor (EGFR) tyrosine kinase inhibitor (TKI) limited its clinical benefit on non-small cell lung cancer (NSCLC). Upfront combination therapy is promising to prevent this resistance. Compelling clinical evidence indicated the failure of third-generation EGFR TKIs combined with either immunotherapy or antiangiogenic agents. In comparison, combined treatment of third-generation EGFR TKIs and chemotherapy might be a favorable choice. Herein, we systematically analyzed and compared the effects of pemetrexed and a novel third-generation EGFR TKI aumolertinib combined in different sequences, ﻿subsequently revealed the potential mechanisms and proved the optimal combination ﻿schedule with clinical retrospective study.

**Methods:**

Three combination schedules involving pemetrexed and aumolertinib in different sequences were developed. Their inhibition effects on cell proliferation and metastasis were firstly compared upon three human NSCLC cell lines in vitro, by cell counting kit-8, colony formation, wound healing and transwell assays respectively. Further evaluation in vivo was proceeded upon H1975 and HCC827 xenograft model. Gene and protein expression were detected by Q-PCR and western blot. Drug concentration was determined by LC–MS/MS. VEGF secretion was determined by ELISA. Tumor vessel was visualized by immunofluorescence. Lastly, a clinical retrospective study was raised with 65 patients’ data.

**Results:**

The combination of pemetrexed and aumolertinib exhibited a sequence-dependent and EGFR mutant-dependent synergistic effect in vitro and in vivo. Only treatment with aumolertinib following pemetrexed (P-A) exhibited synergistic effect with stronger anti-tumor growth and anti-metastasis ability than monotherapy and also other combination sequences. This synergism could exclusively be observed in H1975 and HCC827 but not A549. Pathway analysis showed that P-A significantly enhanced the suppression of EGFR pathway. In addition, our results intriguingly found an obvious reduction of VEGF secretion and the accompanying normalization of the intratumor vessel, consequently increasing intratumoral accumulation of pemetrexed in P-A group. Finally, the clinical retrospective study verified the synergistic effect of P-A combination by significantly superior tumor response than aumolertinib monotherapy.

**Conclusion:**

Aumolertinib-pemetrexed combined therapy is promising for EGFR mutant NSCLC but only in right administration sequence. P-A could become an advantageous combination strategy in clinical with synergistic inhibition of tumor growth and metastasis.

**Supplementary Information:**

The online version contains supplementary material available at 10.1186/s13046-022-02369-3.

## Background

Given that more than 50% of Asian and 10–20% of European non-small cell lung cancer (NSCLC) patients harbored active EGFR mutant, such as deletion mutation in exon 19 (del19) and the point mutation in exon 21 (L858R), mutant EGFR has become a vital therapeutic target for NSCLC [1, 2]. However, most patients with del19 or L858R who show initial clinical responses ultimately developed acquired drug resistance, and 60% of the resistance was caused by secondary T790M mutations [3]. To overcome this major resistance mechanism, the third-generation EGFR TKIs including osimertinib and aumolertinib have been developed and approved for NSCLC patients with EGFR positive or concomitant T790M mutations. Unfortunately, patients inevitably develop a secondary resistance, although third-generation EGFR TKIs have shown potent clinical outcomes in initial several months, thus limiting a prolonged clinical benefit [4, 5].

Emerging evidence reveals that the occurrence of resistance to third-generation EGFR TKIs is associated with long-term drug administration and coinstantaneous selection of pre-existing resistance clones as well as the evolution of drug-tolerant presisters [6, 7], accordingly upfront combination therapy based on different targets and mechanisms is promising to prevent and overcome the resistance to third-generation EGFR TKIs by enhancing tumor cytotoxicity and concomitantly reducing pre-existing resistance clones [8]. Several clinical trials, focusing on third-generation EGFR TKIs-based combination therapy with immune checkpoint inhibitors (ICIs) and antiangiogenic therapy, were performed in recent years [9, 10]. Unfortunately, due to the inhibition of antitumor immune response by EGFR signaling, ICIs treatment of NSCLC patients with EGFR alterations failed to show clinical benefits, especially for those with T790M mutation [11, 12]. Meanwhile, the combination arm of bevacizumab with osimertinib also exhibited negligible prolongation of PFS in patients with EGFR T790M mutation [13, 14]. Moreover, the combination of third-generation EGFR TKI with ICIs and antiangiogenic agents further induced serious safety problems, including the high incidence of interstitial lung disease for ICIs combination [9, 10], as well as the shorter time to treatment failure and high incidence of proteinuria and hypertension during antiangiogenic therapy combination [13]. Consequently, more effective and tolerant third-generation EGFR TKIs-based combination strategies are urgently warranted.

Even though multiple treatments are recommended for NSCLC patients, chemotherapy is still the main modality for NSCLC patients[15, 16]. With respect to chemotherapy, pemetrexed is regarded as the preferred drug for advanced NSCLC as neoadjuvant and adjuvant therapy for its therapeutic benefit and good tolerability [17, 18], even in patients with brain metastases [19].﻿ Recently, clinical evidence further revealed the potential of pemetrexed-EGFR TKIs combination. Combined pemetrexed and the first-generation EGFR TKIs demonstrated an excellent survival benefit [20]. However, this benefit was only limited in EGFR sensitive mutant patients and there was no great advantage in progression-free survival (PFS) and median overall survival (mOS) for patients with acquired T790M mutation, mainly due to the inapplicability of the first-generation EGFR TKIs on T790M mutation [21, 22]. Accordingly, the trials of third-generation EGFR TKI combined with pemetrexed are recommended, while limited preclinical and clinical outcomes can be found. And neither the NCCN guidelines (Version:2.2022, https://www.nccn.org/guidelines/category_1) nor the CSCO guidelines (Version:2021, http://www.csco.ac.cn/) has a clear recommendation about the third-generation EGFR TKI-based combination with chemotherapy yet.

Aumolertinib is a novel, effective and well-tolerated third-generation EGFR TKI, which was marketed in 2020 [23]. Akin to the previously marketed third-generation EGFR TKI osimertinib, aumolertinib is a pyrimidine-based small molecule but further optimized with a cyclopropyl group replacing a methyl group on the indole ring of osimertinib, thus allowing potentially both higher selectivity against EGFR T790M and higher penetration through the blood–brain barrier [24, 25]. In this paper, we performed a systemic assay to determine the effect of pemetrexed and aumolertinib combination. Intriguingly, we found that the synergistic effect of pemetrexed and aumolertinib was sequence-dependent and EGFR mutation-dependent, which was proved by in vitro and in vivo assays, as well as a clinical retrospective study. In addition, we also identified the underlying mechanism of synergistic effect with the enhanced inhibition of intracellular transduction pathways and also the increased intratumor drug accumulation via indirect vascular normalization.

## Methods

### Drugs and Reagents

Commercially available pemetrexed and aumolertinib were both supplied by Hansoh Pharmaceutical Group Co., Ltd. (Shanghai, China). Osimertinib was purchased from AstraZeneca Pharmaceuticals (USA). Cell counting kit-8 was purchased from Beyotime Biotechnology (Beijing, China). Rabbit-monoclonal-antibody against EGFR (ab52894), p-EGFR (ab40815), AKT (ab179463), p-AKT (ab38449), ERK1/2 (ab184699), p-ERK1/2 (ab201015), PARP1 (ab191217), cleaved-PARP1 (ab32064), cleaved-Caspase3 (ab2302), GAPDH (ab8245) and Goat pAb to Rb IgG HRP (ab6721) were purchased from Abcam (USA). Rabbit-monoclonal-antibody against α-SMA, mouse-monoclonal-antibody against CD31, CY2 and CY5- IgG HRP were purchased from Jackson ImmunoResearch (USA). Human HIF-1α, VEGF, TGF-β, ANG, SFLT and Angiostatin primers were synthesized by Introvigen (USA). All other reagents were of analytical grade and commercially available.

### Cell lines

Human NSCLC cell lines A549, H1975 and HCC827 were obtained from Cell bank of Chinese Academy of Sciences (Shanghai, China). The above cell lines were all grown in RPMI 1640 (Gibco, USA) supplemented with 10% fetal bovine serum (FBS; Gibco, USA), penicillin (100 U/ml) and streptomycin (100 μg/ml) at 37 °C in a humidified atmosphere with 5% CO_2_. Human umbilical vein endothelial cell (HUVEC) was obtained from Promocell (Heidelberg, Germany) and cultured in Dulbecco’s modified Eagle’s medium (DMEM; Gibco, USA) supplemented with 10% FBS, 100 U/mL penicillin and 100 mg/mL streptomycin at 37 °C in a humidified atmosphere with 5% CO_2_.

### *Animals and *in vivo* treatment*

Healthy female Balb/c nude mice (16–18 g and 4–5 weeks of age) were obtained from the Beijing Vital River Laboratory Animal Technology Co., Ltd. (Beijing, China). The mice were maintained under a controlled environment (22–24 ℃, 50–60% humidity, 12-h light/12-h dark cycle) with ad libitum access to standard laboratory food and water. H1975 and HCC827 cells (5 × 10^6^ cells in 100 μl sterile PBS) were injected subcutaneously into the left flank of each mouse, respectively. After tumor formation, the mice bearing H1975 or HCC827 subcutaneous tumor were randomly assigned to different groups.

To compare the difference of the in vivo therapeutic efficacy among different combination strategies, the mice bearing H1975 or HCC827 subcutaneous tumor were randomly assigned to the following four groups over several cycles (4 days per cycle): (a) control group: saline (0.9% w/v, *i.p.*, *qd*) and CMC-Na (0.5% w/v, *i.g.*, *qd*); (b) P + A group: concurrent administration of pemetrexed (100 mg/kg, *i.p., qd*) and aumolertinib (20 mg/kg, *i.g., qd*) on day 1; (c) P-A group: pemetrexed (100 mg/kg, *i.p., qd*) administered on day 1 and aumolertinib (20 mg/kg, *i.g., qd*) on day 2; (d) A-P group: aumolertinib (20 mg/kg, *i.g., qd*) administered on day 1 and pemetrexed (100 mg/kg, *i.p., qd*) on day 2. The whole process was repeated five times for HCC827 tumor bearing mice and six times for H1975 tumor bearing mice, respectively. Tumor volume was measured every other day, and the tumor volume was calculated as V = (width* width* length)/2. At the end of the experiment, mice were sacrificed and tumor from each mouse was photted.

To evaluate the in vivo synergistic therapeutic efficacy for P-A sequence treatment, the mice bearing H1975 or HCC827 subcutaneous tumor were randomly assigned to the following five groups and administered over several cycles (4 days per cycle): (a) control group: CMC-Na (0.5% w/v, *i.g.*, *qd*) for 3 successive days following saline (0.9% w/v, *i.p., qd*) on day 1; (b) pemetrexed group: CMC-Na (0.5% w/v, *i.g.*, *qd*) for 3 successive days following pemetrexed administration (100 mg/kg, *i.p., qd*) on day 1; (c) aumolertinib group: aumolertinib (20 mg/kg, *i.g., qd*) for 3 successive days following saline (0.9% w/v, *i.p., qd*) on day 1; (d) P-A group: aumolertinib (20 mg/kg, *i.g., qd*) for 3 successive days following pemetrexed administration (100 mg/kg, *i.p., qd*) on day 1; (e) osimertinib group: osimertinib (20 mg/kg, *i.g., qd*) for 3 successive days following saline (0.9% w/v, *i.p., qd*) on day 1; the whole process was repeated five times for HCC827 tumor bearing mice and six times for H1975 tumor bearing mice, respectively. Tumor volume was measured every other day, and the tumor volume was calculated as V = (width* width* length)/2. At the end of the experiment, mice were sacrificed. Tumor from each mouse was photted, weighted and then collected for further experiments.

For the pharmacokinetic assay, pemetrexed (100 mg/kg, *i.p.*) or aumolertinib (20 mg/kg, *i.g.*) was administered at the next day following repeated cycling drug treatment described above. Blood samples were collected at 0.083, 0.25, 0.5, 1, 2, 4, 6, 8,16 and 24 h post drug administration (blood samples were collected no more than 3 times per mouse). At 4 h and 24 h post drug administration, mice were scarified and tumors were collected. The concentrations of pemetrexed and aumolertinib in blood sample or tumor mass were determined by LC–MS/MS.

### Synergistic effect of pemetrexed and aumolertinib on cell growth inhibition

A549, HCC827 and H1975 were seeded in 96-well plates (10,000 cells per well) and exposed to serial dilutions of aumolertinib or pemetrexed for 72 h. For A549, the series concentrations of pemetrexed were 0.001, 0.01, 0.05, 0.1, 0.5, 2, 10, 50 μM and the series concentrations of aumolertinib were 1, 2, 5, 10, 20, 50, 100 μM. For HCC827, the series concentrations of pemetrexed were 0.001, 0.01, 0.1, 0.5, 2, 10, 200 μM and the series concentrations of aumolertinib were 0.0003, 0.001, 0.003, 0.01, 0.03, 0.1, 0.3, 1 μM. For H1975, the series concentrations of pemetrexed were 0.001, 0.01, 0.05, 0.5, 2, 10, 200 μM and the series concentrations of aumolertinib were 0.01, 0.1, 1, 10, 20, 50, 100 μM. After treatments, cell viabilities were measured by a CCK-8 Assay Kit (KeyGEN BioTech, Nanjing, China) and quantified relatively to those in wells without drugs. IC_50_ values were calculated from inhibition curves using GraphPad Prism 8.

Three different combination strategies were designed as follows: (a) P + A: pemetrexed and aumolertinib were co-administered simultaneously for 72 h; (b) P-A: pemetrexed for 24 h previously, and followed by aumolertinib for another 72 h; (c) A-P: aumolertinib for 24 h previously, and followed by pemetrexed for another 72 h. Integration effects of these combination strategies on A549, HCC827 and H1975 were evaluated and compared using combination index (CI). During the experiment, three NSCLC cell lines were treated with series concentrations of pemetrexed and aumolertinib at the ratio of their natural IC_50_ values, respectively. For A549, the series concentrations of pemetrexed were 1.25 μM (0.25*IC_50_), 2.5 μM (0.5*IC_50_), 5 μM (1*IC_50_), 10 μM (2*IC_50_), 20 μM (4*IC_50_), the series concentrations of aumolertinib were 5 μM (0.25*IC_50_), 10 μM (0.5*IC_50_), 20 μM (1*IC_50_), 40 μM (2*IC_50_), 80 μM (4*IC_50_). For HCC827, the series concentrations of pemetrexed were 0.038 μM (0.25*IC_50_), 0.076 μM (0.5*IC_50_), 0.152 μM (1*IC_50_), 0.304 μM (2*IC_50_), 0.608 μM (4*IC_50_), the series concentrations of aumolertinib were 0.01 μM (0.25*IC_50_), 0.02 μM (0.5*IC_50_), 0.04 μM (1*IC_50_), 0.08 μM (2*IC_50_), 0.16 μM (4*IC_50_). For H1975, the series concentrations of pemetrexed were 0.078 μM (0.25*IC_50_), 0.156 μM (0.5*IC_50_), 0.312 μM (1*IC_50_), 0.625 μM (2*IC_50_), 1.25 μM (4*IC_50_), the series concentrations of aumolertinib were 0.312 μM (0.25*IC_50_), 0.625 μM (0.5*IC_50_), 1.25 μM (1*IC_50_), 2.5 μM (2*IC_50_), 5 μM (4*IC_50_). Cell viabilities were measured by a CCK-8 Assay. Raw data obtained for the effects of monotherapy and different combination strategies were entered in online software ComboSyn (http://www.combosyn.com) to obtain model parameter, CI and concentration-effect plots. In our study, CI < 0.75 indicated synergistic effect, 0.75 < CI < 1.45 indicated additive effect and CI > 1.45 indicated antagonism effect.

### Wound healing assay

Cell migration was assessed in a classical wound healing assay with some minor modifications. Briefly, cells were seeded in 6-well plates and the cell layer was gently wounded using a plastic pipette tip after 90%-100% cell confluence. The bottoms of the wells were marked to indicate where the initial images of the wounded area were captured. And the crosses of wounding lines and horizontal lines were observed at different time points (0, 24, 48 h) by Lionheart FXTM Intelligent Live Cell Imaging Analysis System (Bio-Tek Instruments, USA). The wound gaps were measured by Image J software. The migration rate was calculated as follows: migration rate = (wound gap (0 h)—wound gap (48 h)) /wound gap (0 h). Wound gap = wound area/wound length.

### Transwell migration and invasion assay

For cell migration assay, 1 × 10^5^ HCC827 or H1975 were added to the upper chambers directly, and for the cell invasion assay, 2 × 10^5^ HCC827 or H1975 were added to the upper chambers pre-coated with Matrigel. After incubation for 18 h, the upper chambers were rinsed with ice-cold PBS, fixed with 4% paraformaldehyde for 10 min and stained with 0.1% crystal violet. Then, the chambers were washed thoroughly in running water and the cells which didn’t migrate through pores were wiped off with cotton swabs. Images were taken with microscope in bright field and the number of cells was measured by Image J software.

### Western blot

The immunoblotting assays were compiled as described previously [26]. Cell samples or tumors were lysed on ice with homogenizer in NP40 buffer supplemented with 100 μM Phenylmethanesulfonyl fluoride and 0.1% (v/v) phosphatase inhibitor (Beyotime Biotechnology, China). Protein was extracted by centrifugation (10,000 g, 5 min, 4 ℃). Protein concentrations were determined by the bicinchoninic acid (BCA) Protein Assay. Equal amounts of protein (30 μg) were loaded for each lane, separated by 8%, 10% or 12% SDS-PAGE gel and transferred to PVDF membranes (Bio-Rad, USA). After the transfer, the blots were first saturated by incubation in 5% skim milk (in 10 mM Tris–HCl containing 150 mM sodium chloride and 0.5% Tween 20) for 1 h at 37 ℃ and then incubated overnight at 4 ℃ with antibodies against EGFR (1:1000, Abcam, Cat#ab52894), p-EGFR (1:1000, Abcam, Cat#ab40815), AKT (1:10,000, Abcam, Cat#ab179463), p-AKT (1:500, Abcam, Cat#ab38449), ERK1/2 (1:10,000, Abcam, Cat#ab184699), p-ERK1/2 (1:1000, Abcam, Cat#ab201015), PARP1 (1:1000, Abcam, Cat#ab191217), cleaved-PARP1 (1:1000, Abcam, Cat#ab32064), cleaved-Caspase3 (1:500, Abcam, Cat#ab2302), GAPDH (1:4000, Abcam, ab8245). These blots were further incubated with Goat pAb to Rb IgG HRP (1:10,000, Abcam, ab6721) for 1 h at 37 ℃, developed in ECL solution, and visualized using an enhanced chemiluminescence detection kit and captured using a ChemiDoc XRS − System (Bio-Rad, USA). Signal intensities were normalized to GAPDH. The intensity of the selected band was analyzed using ImageJ.

### Immunofluorescence

Xenograft tumor tissues were collected and fixed overnight in 4% paraformaldehyde and then dehydration with 20% and 30% sucrose, respectively. Tumor tissues were cut into 10 μm sections (free-floating) in a cryostat and processed for immunofluorescence as previously described [27]. To determine the vessel branches and calculate the tumor microvascular density, the sections were incubated with anti-CD31 (1:100, BD Biosciences, Franklin Lakes, NJ, USA) and α-SMA (1:100, BD Biosciences, Franklin Lakes, NJ, USA) at 4 ℃ overnight and then incubated with Cy5- or Cy2-conjugated secondary antibody (1:200, Jackson ImmunoResearch, West Grove, PA, USA) for 1 h at 37 ℃. The stained sections were observed with confocal microscope (Olympus FV3000). Cy2 was determined at excitation wavelength 489 nm and emission wavelength 506 nm, Cy5 was determined at excitation wavelength 650 nm and emission wavelength 670 nm. α-SMA^+^ or CD31^+^ area was measured by Image J software.

### Real-time quantitative PCR

Total RNA of cell samples or tumors was extracted using a High Pure RNA Isolation Kit (RNAiso Plus, Takara, Japan) and reverse transcribed using a PrimeScript RT Regent Kit (Vazyme, Nanjing, China). mRNA expression was assessed by RT-quantitative PCR using a CFX96 real-time detection system (Bio-Rad, USA). The cycling conditions were as follows: 95 ℃ for 10 min, followed by 40 cycles with 95 ℃ for 15 s, 60 ℃ for 30 s, and 72 ℃ for 30 s. Melting curve analysis was performed routinely to verify the specificity of real-time PCR products. Specific mRNA values were calculated after normalization of the results for each sample with those for β-actin mRNA. The data are presented as relative mRNA units with respect to control values. Quantification was performed by the comparative Ct method (2^△△Ct^: normalizing cycle threshold (Ct) values with β-actin Ct). The gene-specific primers used in this study are shown in Table S1.

### VEGF determination by ELISA

Thirty mg tumor tissue was homogenized with homogenizer in 300 μl pure water. Tumor tissue homogenates were diluted 1:50 in assay diluent solution. The VEGF levels in tumor tissue homogenates and cell supernatants were measured using the human VEGF ELISA kit (ExCell Bio, Shanghai, China) according to the manufacturer’s protocol. Absorbance was measured at 450 nm after the addition of stop solution.

### LC–MS/MS-based quantitative analysis of pemetrexed and aumolertinib

The concentrations of pemetrexed and aumolertinib in the plasma, tumor and other tissues were all analyzed on a Shimadzu LC-10AD HPLC system (Kyoto, Japan) coupled to API 4000 (SCIEX, Birmingham, MA, USA). Briefly, plasma and tissue homogenates were protein-precipitated with 3 times volume of ice-cold methanol containing 500 ng/ml osimertinib (Internal Standard, IS). After twice centrifugation (30,000 g, 10 min, 4 °C), the supernatant was injected into the LC–MS/MS system for analysis.

For analysis of pemetrexed and aumolertinib, chromatographic separation was performed on a ZORBAX Eclipse Plus C18 column (150 × 4.6 mm, 5 μm, Agilent, USA) at 40 °C. The mobile phase consisted of solvent A (0.1% acetic acid and 5 mM ammonium acetate) and solvent B (acetonitrile) with the following gradient: 1 min, 1% B; 5 min, 70% B; 8 min, 70% B; 9.5 min, 1% B; 12 min, 1% B. The flow rate was 0.7 ml/min. The mass spectrometer was operated in positive electrospray ionization (ESI) mode. The multiple rection monitoring (MRM) parameters were set as follows: declustering potential set at 80 V for pemetrexed and osimertinib and 70 V for aumolertinib, collision energy set at 27 eV for pemetrexed, 33 eV for aumolertinib and 30 eV for osimertinib, MRM transition set as m/z 428.1 → 281.2 for pemetrexed and m/z 526.5 → 481.3 for aumolertinib and m/z 500.8 → 455.3 for osimertinib.

### Clinical retrospective study

We screened patients in Department of Respiratory Medicine (The Affiliated Brain Hospital of Nanjing Medical University) who had received aumolertinib as first-line therapy from April, 2020 to January, 2022. Eligibility for evaluation within the retrospective study was based on the diagnosis of computed tomography, pathologic evaluation and gene detection. Only patients with primary NSCLC harboring EGFR mutant were involved in the study. Overall, 65 patients were submitted, among which 50 patients received aumolertinib monotherapy and 15 patients received combination therapy. For combination therapy, patients first used pemetrexed/cisplatin on day 1 and following aumolertinib on day 8–28 in a 28-day cycle for up to 2 cycles. Tumor response was evaluated by computed tomography scans according to the Response Evaluation Criteria in Solid Tumor Criteria Version 1.1. Complete response (CR) means disappearance of all target lesions. Partial response (PR) means that the longest diameter of target lesion was reduced by at least 30%. Progressive disease (PD) means that the longest diameter of the target lesion increases by at least 20%, or the appearance of new lesion. Stable disease (SD) means that the longest diameter of the target lesion increased to less than PD, or reduced to less than PR. Disease control rate (DCR) = (CR + PR + SD) / total number of cases, and the objective response rate (ORR) = (CR + PR) / total number of cases.

For five representative cases who used the combination of aumolertinib and chemotherapy as neoadjuvant therapy, pathological response was assessed by local pathologists, who measured the percentage of residual viable tumor in primary tumors resected from each patient during surgery. Tumors with < 10% viable tumor cells were considered to have a major pathologic response (MPR) and those with no viable tumor cells were deemed to be complete pathological response (CPR).

### Data analysis

For preclinical study, all data are presented as mean ± standard error of mean (SEM). Statistical analyses were performed using GraphPad Prism 8 software. Each continuous variable was analyzed for a normal distribution using the Kolmogorov–Smirnov test, and then statistical analysis was performed using a two-tailed Student’s t-test or one-way ANOVA assay with Dunnett post-hoc test if F was less than 0.05 and there was no significant variance inhomogeneity. Differences were considered significant at *p < 0.05, **p < 0.01, ***p < 0.001.

For clinical study, data are median or n (%). p values were calculated by Mann–Whitney test. Differences were considered significant at *p < 0.05.

## Results

### The combination of pemetrexed and aumolertinib exhibits a sequence-dependent synergistic effect in EGFR-mutant NSCLC cell lines

**Fig. 1:**
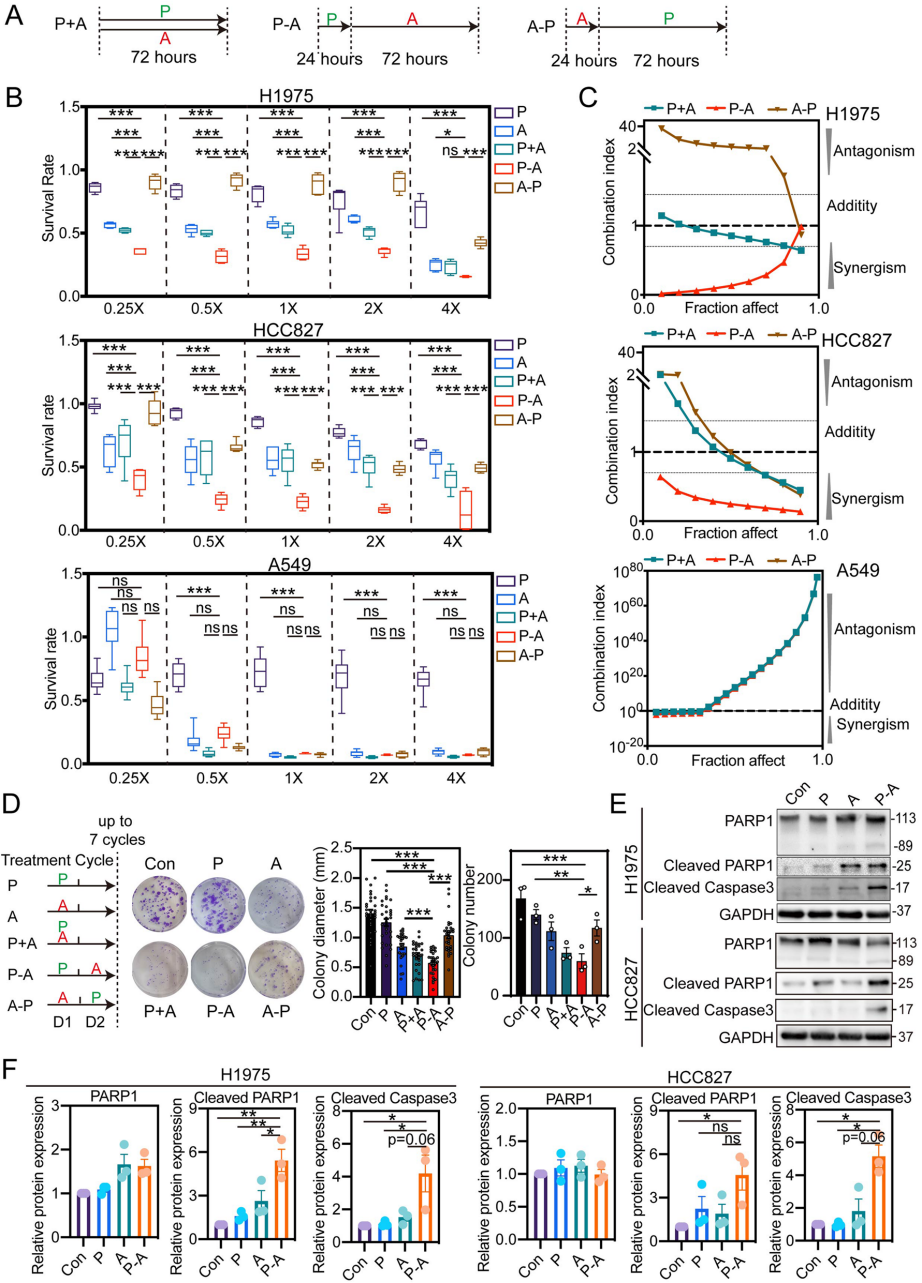
Sequence-dependent synergistic effect on tumor cell growth inhibition in EGFR-mutant NSCLC cell lines. **A** Schematic presentation of three different combination strategies. **B** Three NSCLC cell lines, including EGFR L858R/T790M H1975, EGFR-del19 HCC827 and EGFR-wide type A549, were exposure to different combination strategies of pemetrexed and aumolertinib or alone with serious doses at constant ratios of the IC_50_ (*n* = 6). **C** Combination indexes (CI) of different combination mode were evaluated in three NSCLC cell lines. The CI value > 1.45 represents antagonistic effect, 0.7 < CI < 1.45 represents addictive effect while CI < 0.7 represents synergistic effect (*n* = 6). **D** Representative images of colony formation assay of HCC827 after different drug treatment. 20 μM pemetrexed and 20 nM aumolertinib were used for HCC827 (*n* = 3). **E** A western bolt assay was performed to detect the changes of PARP1, cleaved PARP1 and cleaved Caspase 3 expression in H1975 and HCC827 cell lines. In this experiment, 20 μM pemetrexed and 2 μM aumolertinib were used for H1975. 20 μM pemetrexed and 20 nM aumolertinib were used for HCC827. **F** Quantification of the western blot band intensity was performed using ImageJ and GAPDH was used as loading controls (*n* = 3). Con represents control; P represents pemetrexed; A represents aumolertinib; P + A represents concomitant treatment with pemetrexed and aumolertinib; P-A represents pemetrexed treatment followed by aumolertinib treatment; A-P represents aumolertinib treatment followed by pemetrexed treatment. All of the data were expressed as the mean ± SEM, ns represents no significance, * *p* < 0.05, ** *p* < 0.01 and *** *p* < 0.001

We initially determined the IC_50_ values of pemetrexed or aumolertinib on different NSCLC cell lines including EGFR-wide type A549, EGFR-del19 HCC827 and EGFR-L858R/T790M H1975 (Fig. S1). And then three different combination strategies were designed (Fig. 1A). The integration index of each combination strategy was measured with various concentrations of pemetrexed and aumolertinib at the ratios of their natural IC_50_ values, ranging from 0.25 times IC_50_ concentration to 4 times IC_50_ concentration. As shown in Fig. 1B, P-A was a superior strategy in H1975 and HCC827, with significantly stronger inhibitory effects on survival rates than single drug treatment and also other combination strategies. Furthermore, the CI value showed that only the anti-proliferative effect of the strategy P-A resulted in synergy (CI < 0.75) at all concentration points in both H1975 and HCC827 (Fig. 1C). In comparison, strategy P + A just generated additive effect (0.75 < CI < 1.45) at all concentration points in H1975 as well as high concentration in HCC827, and even antagonistic effect (CI > 1.45) at low concentration points in HCC827. The most inferior strategy A-P, generated antagonistic effect at all concentration points in H1975 and high concentration in HCC827 with only additive effect at low concentration points in HCC827. However, different from EGFR-mutant cell lines, all combination strategies exhibited antagonistic effect at all concentration points in EGFR wide type cell line A549. Next, this sequence-dependent synergistic effect of proliferative inhibition upon EGFR-mutant NSCLC cell lines were further validated by colony formation analysis (Fig. 1D). In addition, apoptosis plays a crucial role in the response of cancer to EGFR TKIs [7] and also pemetrexed [28]. Our results showed ﻿that pro-apoptosis effect markedly enhanced post P-A sequence treatment, revealed by significantly increased expression of cleaved Caspase 3 and cleaved PARP1 (Fig. 1E-F).

**Fig. 2:**
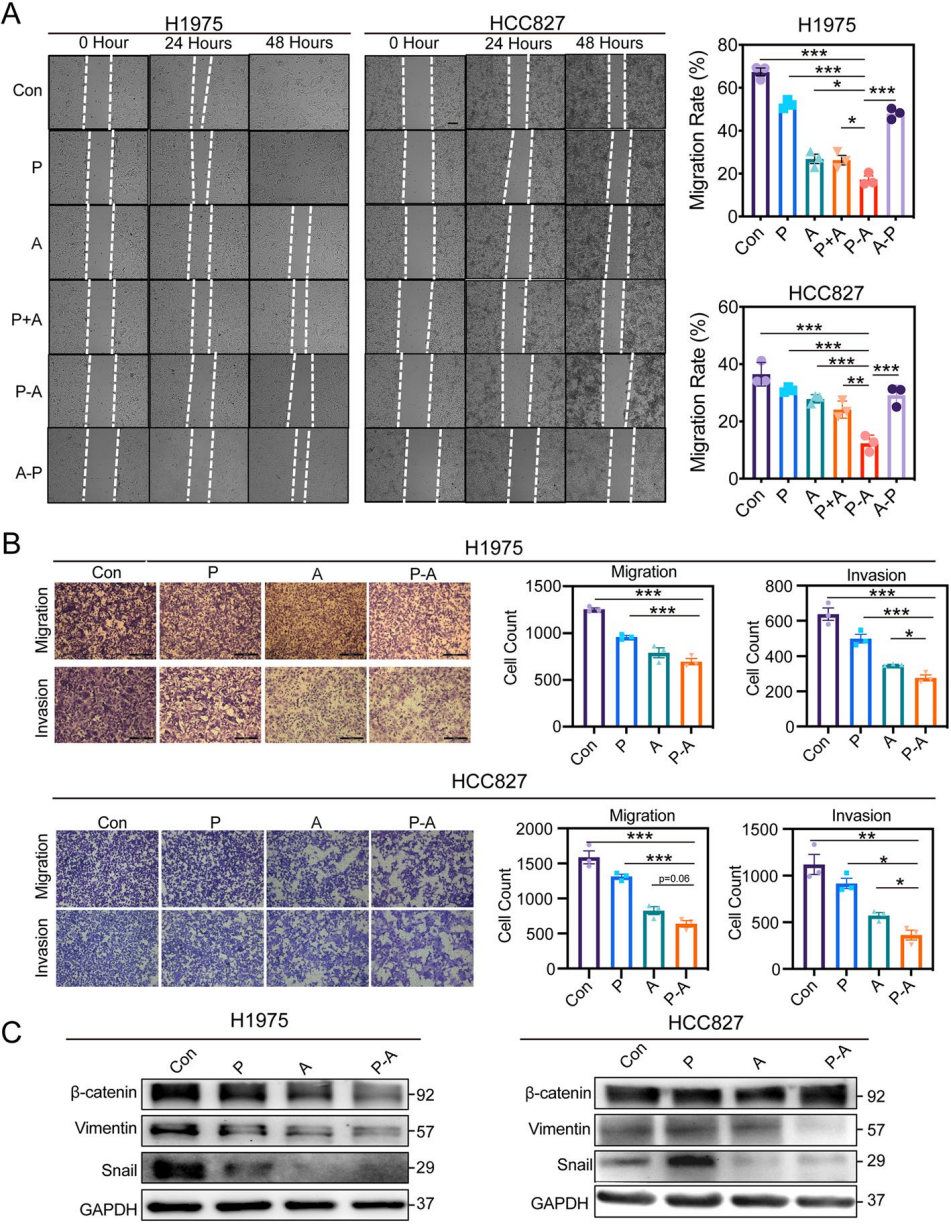
Sequence-dependent synergistic effect on tumor cell metastasis inhibition in EGFR-mutant NSCLC cell lines. **A** Wound healing assays were performed in H1975 and HCC827 to determine the effects of different drug treatments on cell migration.10 nM pemetrexed and 10 nM aumolertinib were used for H1975. 5 nM pemetrexed and 5 nM aumolertinib were used for HCC827. The crosses of wounding lines and horizontal lines were observed at 0 h, 24 h and 48 h post drug administration. The cell migration rates were quantified and compared (*n* = 3). **B** Transwell assays were further performed in H1975 and HCC827 to prove the synergetic effect of P-A on tumor metastasis inhibition. 10 nM pemetrexed and 10 nM aumolertinib were used for H1975. 5 nM pemetrexed and 5 nM aumolertinib were used for HCC827. Representative images were shown and the average number of migrating or invasive cells in each group was counted (*n* = 3). **C** The EMT-related proteins, β-catenin, vimentin and snail, were analyzed by western blot after P-A sequence treatment or single drug treatment. Con represents control; P represents pemetrexed; A represents aumolertinib; P + A represents concomitant treatment with pemetrexed and aumolertinib; P-A represents pemetrexed treatment followed by aumolertinib treatment; A-P represents aumolertinib treatment followed by pemetrexed treatment. All of the data were expressed as the mean ± SEM, * *p* < 0.05, ** *p* < 0.01 and *** *p* < 0.001

Similar to the antiproliferative effect, P-A also exhibited superior inhibition on cell migration and invasion. The migration rate of H1975 and HCC827 after P-A treatment was only 17.24% and 12.39%, significantly lower than the single drug treatment groups. In comparison, P + A and A-P treatment failed to enhance the inhibition effect of aumolertinib on cell migration (Fig. 2A). Transwell migration and invasion assay further validated the synergistic effect of pemetrexed and aumolertinib combination at P-A sequence (Fig. 2B). Moreover, we examined several factors involved in EMT since the EMT is a crucial step for EGFR-induced tumor metastasis. As shown in Fig. 2C, β-catenin, vimentin and snail were significantly reduced after drug treatments, among which, P-A exhibited the strongest inhibition.

### The combination of pemetrexed and aumolertinib exhibits a sequence-dependent synergistic effect in EGFR-mutant NSCLC bearing mice

**Fig. 3:**
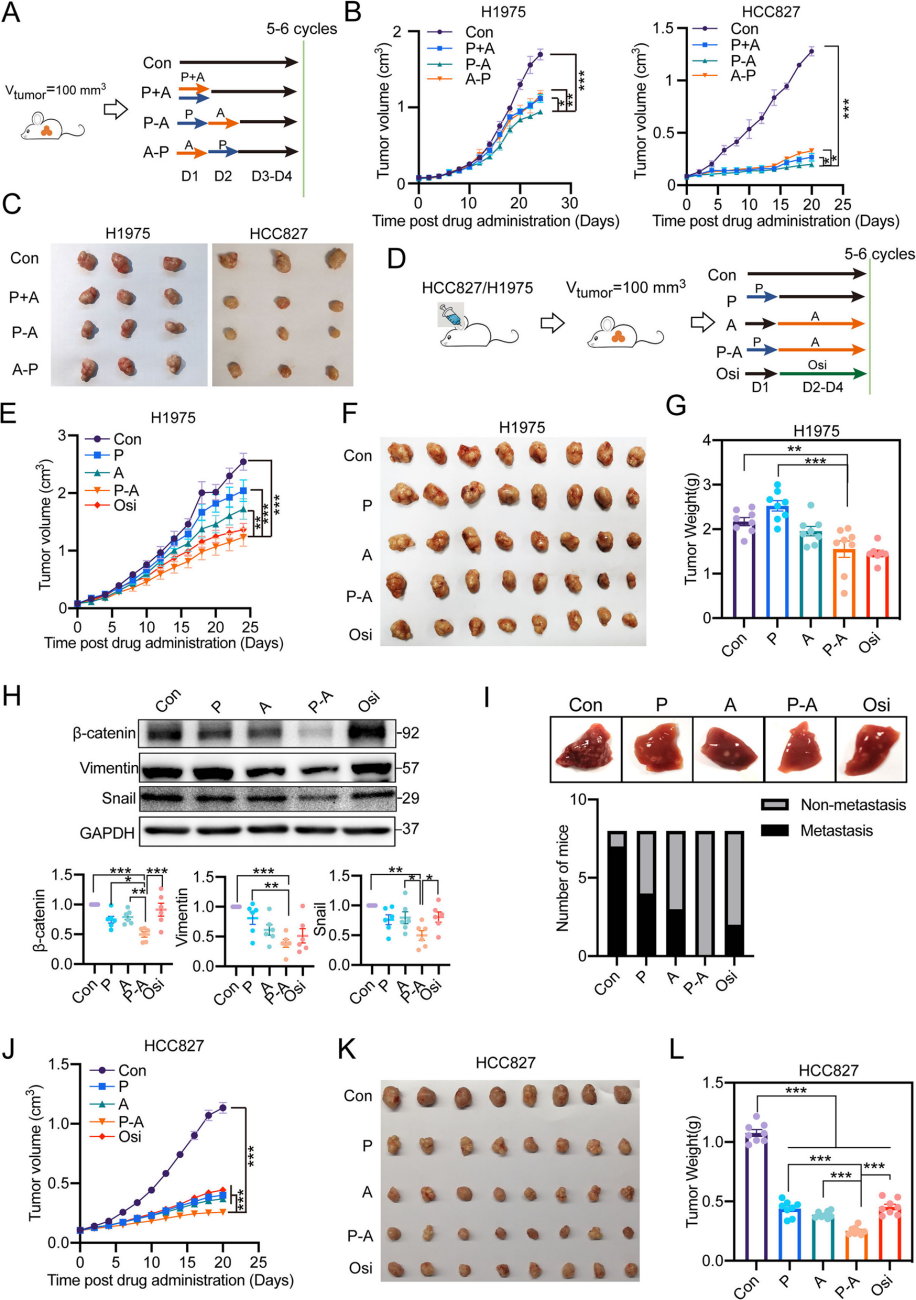
Sequence-dependent synergistic effect on tumor growth and metastasis inhibition in EGFR-mutant NSCLC bearing mice. **A **A small-scale in vivo experiment (*n* = 3) was designed to compare the anti-tumor effects of different combination sequences. **B** Tumor volumes were measured every other day and tumor growth curves were plotted for mice bearing H1975 or HCC827 cell-derived tumor xenografts (*n* = 3). **C** At the endpoint of drug administration, mice in each group were sacrificed, and the tumors were dissected and photographed (*n* = 3). **D** Another systemic in vivo experiment (*n* = 8) was proposed to determine the advantage of P-A sequence over single drug administration. Schematic presentation of different administration strategies in H1975 and HCC827 tumor-bearing mice was shown. **E** Volumes of H1975 cell-derived tumor xenografts were measured every other day and tumor growth curves were plotted against time (*n* = 8). **F** H1975 tumor-bearing mice were sacrificed post 6-cycle drug administration, and the tumors were dissected and photographed (*n* = 8). **G** The weight of H1975 cell-derived tumor xenografts at the endpoint were analyzed and compared (*n* = 8). **H** Intratumoral expression of EMT-related protein, β-catenin, vimentin and snail, were analyzed by western blot. Quantification of the western blot band intensity was performed using ImageJ and GAPDH was used as loading controls (*n* = 6). **I** Metastatic nodule detection in liver was performed. **J** Similar to the experimental operation on H1975 tumor bearing mice, P-A sequence treatment and single drug administration were also applied on HCC827 tumor bearing mice. Tumor volumes were measured every other day and tumor growth curves were plotted (*n* = 8). **K** HCC87 tumor-bearing mice were sacrificed post 5 cycles drug administration, and the tumors were dissected and photographed (*n* = 8). **L** The weight of HCC827 cell-derived tumor xenografts at the endpoint were analyzed and compared (*n* = 8). Con represents control; P represents pemetrexed; A represents aumolertinib; Osi represents osimertinib; P + A represents concomitant treatment with pemetrexed and aumolertinib; P-A represents pemetrexed treatment followed by aumolertinib treatment; A-P represents aumolertinib treatment followed by pemetrexed treatment. All of the data were expressed as the mean ± SEM, * *p* < 0.05, ** *p* < 0.01 and *** *p* < 0.001

Based on the difference of integration effect among various combination strategies in vitro, we hence inferred that P-A would exhibit superior anti-tumor effect over other combination strategies. This hypothesis was confirmed by a small-scale in vivo assay (Fig. 3A). As shown in Fig. 3B, the tumor volume of P-A group was significantly smaller than that of P + A treatment group and A-P treatment group. At the end of drug treatment, tumors were collected and photographed (Fig. 3C). Among all drug treatment groups, the tumor burden of P-A was the lightest, suggesting the superior anti-tumor effect of P-A.

Next, we applied a systemic experiment to determine the synergy of P-A sequence on both H1975 and HCC827 tumor bearing mice (Fig. 3D). As shown in Fig. 3E, the progress of H1975 tumor was slower with drug treatment, among which, P-A exhibited significant anti-tumor effect at the earliest and at the most extent. In comparison of monotherapy treatment and combination drug treatment, we found that the tumor volume of P-A group was significantly smaller than that of pemetrexed treatment group since day 14 and aumolertinib treatment group since day 18, indicating a synergistic effect (Fig. S2A). At the end of drug treatment, tumors were collected (Fig. 3F) and tumor weights were assayed. Among all drug treatment groups, the tumor burden of P-A was lightest (Fig. 3G). Furthermore, in agreement with in vitro assay, P-A exhibited stronger inhibition on tumor metastasis, proved by the obviously reduced expression of β-catenin, vimentin and snail in tumor (Fig. 3H) and lower metastasis in liver (Fig. 3I).

The synergistic effect of P-A sequence was further substantiated by the markedly increased antitumor effect when the treatment strategy was applied to another EGFR-mutant NSCLC bearing mice. In comparison with H1975, HCC827 was more sensitive to drug treatment, in accordance with the smaller IC_50_ assayed in vitro (Fig. S1)*.* As shown in Fig. 3J and Fig. S2B, P-A exhibited a significant anti-tumor effect since day 4 post drug treatment, and both pemetrexed and aumolertinib treatment group showed anti-tumor effect after 6-days drug treatment. Despite the different drug sensitivities between HCC827 and H1975, P-A sequence treatment also exhibited superior anti-tumor effect on HCC827 tumor bearing mice, similar to the phenomenon observed in H1975 tumor bearing mice. The tumor volume of P-A group was significantly smaller than pemetrexed and aumolertinib monotherapy group since day 10. Furthermore, the tumor burden of P-A sequence group was lightest after 5-cycles drug treatment, with only 23.3% volume and 23.4% weight of the control, significantly smaller than both pemetrexed and aumolertinib monotherapy groups (Fig. 3K and L).

During the whole period of drug treatment, the mice in all groups showed no obvious changes of food and water intake, and the body weight kept stable, indicating the safety of drug administration (Fig. S2C).

### P-A sequence improves the suppression of EGFR signaling pathway

**Fig. 4:**
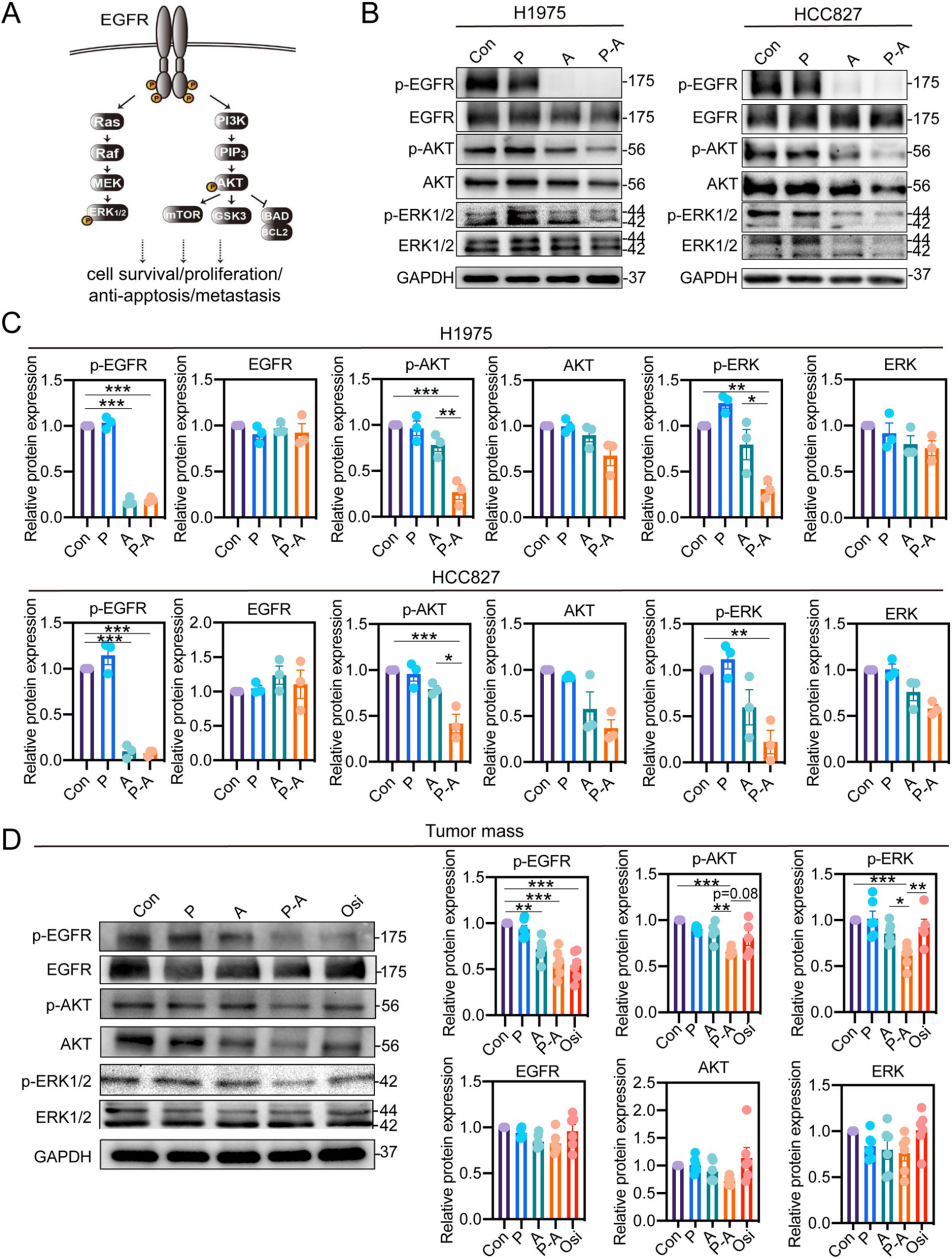
P-A sequence improves the suppression of EGFR signaling pathway**.** **A** Schematic presentation of EGFR signaling pathway. **B** A western blot assay was performed to detect the expression of the representative proteins within EGFR pathway in both H1975 and HCC827 cell line. In this experiment, 20 μM pemetrexed and 2 μM aumolertinib were used for H1975. 20 μM pemetrexed and 20 nM aumolertinib were used for HCC827. **C** Quantification of the western blot band intensity of p-EGFR, EGFR, p-AKT, AKT, p-ERK and ERK in H1975 and HCC827 cell line were performed using ImageJ and GAPDH was used as loading controls (*n* = 3). **D** Furthermore, the changes of EGFR signaling pathway in H1975 tumor mass post different drug administration were also determined and quantified (*n* = 6). Con represents control; P represents pemetrexed; A represents aumolertinib; Osi represents osimertinib; P-A represents pemetrexed treatment followed by aumolertinib treatment. All of the data were expressed as the mean ± SEM, * *p* < 0.05, ** *p* < 0.01 and *** *p* < 0.001

The binding of EGF and dimerization of EGFR, a transmembrane glycoprotein, activates EGFR and the downstream PI3K-AKT and ERK signaling pathway, which can regulate cell survival, proliferation, anti-apoptosis, and metastasis (Fig. 4A). Suppression of EGFR signaling pathway was the crucial mechanism for the anti-proliferation and anti-metastasis effect of EGFR TKIs. Herein, we firstly detected the activation of EGFR and its downstream signaling pathway upon H1975 and HCC827 to gain the insight of the molecular mechanism behind the synergistic effects during P-A sequence treatment. As shown in Fig. 4B and C, we noted a significant inhibition effect on phosphorylating EGFR and concomitantly the reduced expression of p-ERK and p-AKT after aumolertinib or P-A sequence treatment, in contrast pemetrexed showed no effect on EGFR pathway. Interestingly, despite no direct effect of pemetrexed on EGFR signaling pathway, the levels of p-AKT and p-ERK expression in P-A sequence treatment group were much lower than that of aumolertinib monotherapy, which were validated in two EGFR-mutant cell lines. We also tested whether the enhanced suppression of EGFR signaling pathway post P-A sequence treatment can be found in tumor mass. Consistent with the results found in the cell lines, the expression of p-ERK and p-AKT in tumor mass after P-A sequence treatment was significantly lower than that in aumolertinib treatment group (Fig. 4D). However, when sequence alternating, the enhanced suppression of EGFR signaling pathway disappeared after A-P sequence treatment (Fig. S3). Besides, in line with resembled anti-tumor effect, aumolertinib and osimertinib suppressed EGFR signaling pathway to similar magnitude (Fig. 4D and Fig. S4).

### P-A sequence improves the accumulation of pemetrexed in tumor via vascular normalization

**Fig. 5:**
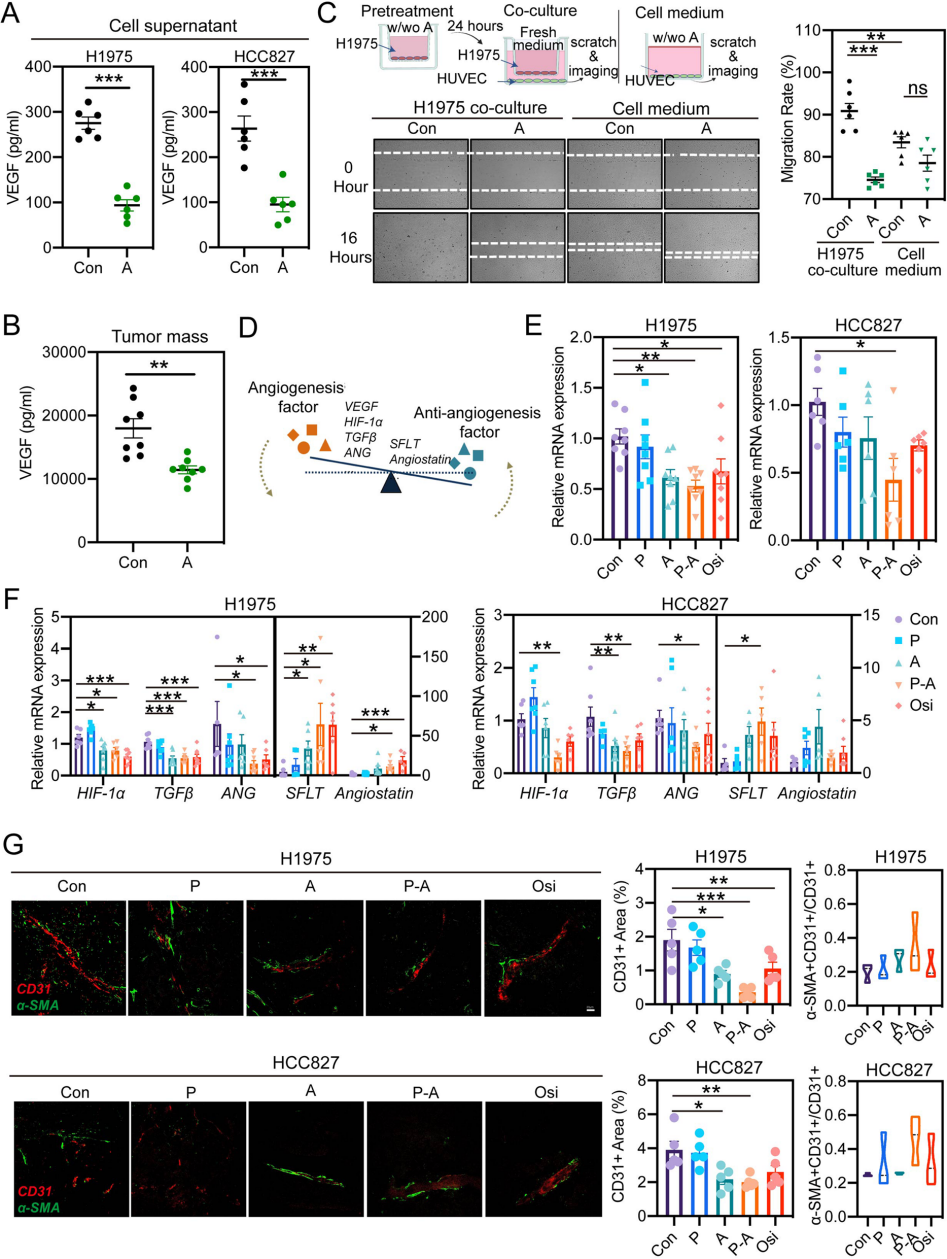
Aumolertinib exerts vascular normalization by decreasing VEGF secretion from EGFR-mutant tumor cells. **A** H1975 and HCC87 were cultured in serum-free media with or without the 2 μM aumolertinib and the VEGF amount in the media was measured by ELISA (*n* = 6). **B** Intratumoral amounts of VEGF amount in control group and Aumolertinib administration group were measured and compared (*n* = 8). **C** H1975 was pre-cultured for 24 h in the absence or presence of 2 μM aumolertinib, and then co-cultured with HUVEC following changing fresh medium. The migrations of HUVEC were monitored and images were shot at 16 h post co-culture. Meanwhile, the migrations of HUVEC were also monitored in the medium with or without aumolertinib to determine the direct influence of aumolertinib on HUVEC (*n* = 6). **D** Schematic presentation of the rebalance of angiogenesis and anti-angiogenesis factor in the tumor mass. Gene expressions of VEGF **E** and other mainly factors **F** in H1975 cell- and HCC827 cell-derived tumor xenografts with different drug treatment were assayed by qPCR (*n* = 8 for H1975 and *n* = 6 for HCC827). Gene expression was normalized to the housekeeping gene β-actin. **G** Endothelium and associated pericytes were visualized by CD31 (red) and α-SMA (green) immunofluorescence staining of H1975 and HCC827 tumor xenograft (*n* = 5). Representative images of different groups were shown. The fraction of CD31^+^ area and the relative proportion of α-SMA^+^ pericyte-covered blood vessels were quantified by image J. Con represents control; P represents pemetrexed; A represents aumolertinib; Osi represents osimertinib; P-A represents pemetrexed treatment followed by aumolertinib treatment. All of the data were expressed as the mean ± SEM, * *p* < 0.05, ** *p* < 0.01 and *** *p* < 0.001

Since the latest report about an altered secretion of VEGF after EGFR activating mutation occurring in NSCLC cells [29], we sought to investigate whether aumolertinib influences the secretion of VEGF from H1975 and HCC827. Figure 5A shows a significant reduction of VEGF secreted into the cell medium after aumolertinib treatment. Similarly, the amount of VEGF was also significantly reduced in the tumor mass after aumolertinib administration (Fig. 5B). The distinct change of VEGF secretion following the suppression of EGFR signaling pathway in NSCLC cells suggested an indirect influence of EGFR TKIs on tumor vessels. Expectedly, the migration of endothelial cell HUVEC was significantly promoted after the co-culture with H1975, while the migration was inhibited when HUVECs were priorly exposed to aumolertinib for 24 h. In addition, this inhibition could not be observed when HUVEC directly exposure to aumolertinib (Fig. 5C). Based on the important role of VEGF on pathological angiogenesis in tumor mass and also the normalization of the abnormal structure and function of tumor vasculature during the treatment with antiangiogenic agents targeting VEGF/VEGFR2 [30], we hence inferred that vascular normalization would occur in tumor mass after EGFR TKIs treatment. This hypothesis was confirmed in Fig. 5D-G with the significant decreases of VEGF and other pro-angiogenic factors (*HIF-1α, TGFβ, ANG*) as well as the obviously increases of anti-angiogenesis factors (*sFLT*, *Angiostatin*) after EGFR TKIs treatment including aumolertinib or osimertinib monotherapy, and P-A sequence combination therapy. Accompanied by the rebalance of pro-angiogenic and anti-angiogenesis factors, the normalization of the abnormal tumor vasculature structure was observed. Double staining of vascular endothelial cells marker CD31 (Red) and smooth muscle cells marker α-SMA (Green) was the indicator of vascular maturity. Figure 5G shows that originally thin, short and clutter tumor vessels in the control group were markedly prolonged and simultaneously exhibited higher co-location of CD31^+^ and α-SMA^+^ post EGFR TKIs treatment. Among three groups involving EGFR TKIs, P-A groups exerted superior effect on vascular normalization, in agreement with the strongest suppression of EGFR signaling pathway.

**Fig. 6:**
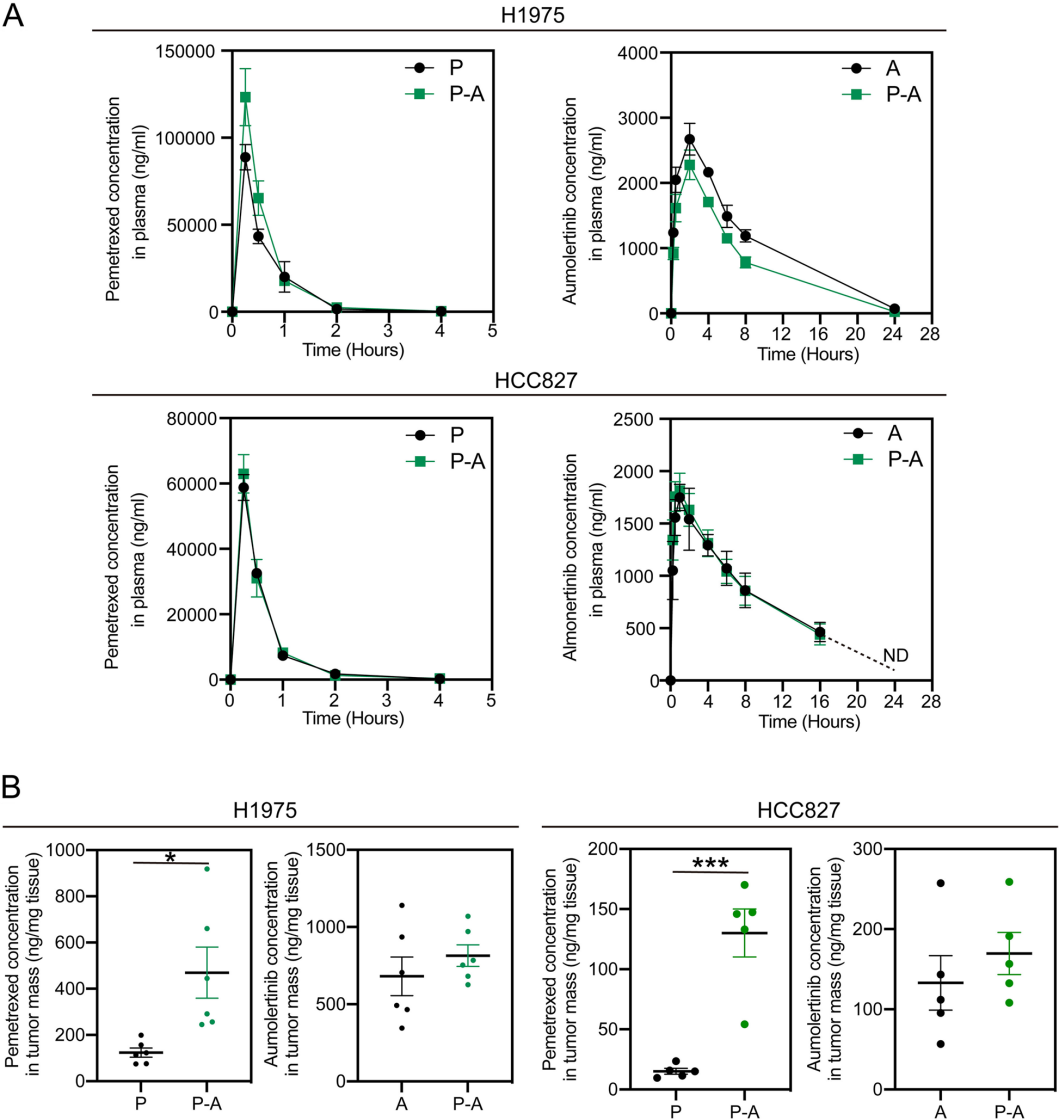
P-A sequence specifically increased the accumulation of pemetrexed in tumor mass. **A** Pharmacokinetic profiles of pemetrexed and aumolertinib in plasma of H1975 and HCC827 tumor bearing mice in the absence and presence of sequence administration were plotted, respective (*n* = 4). **B** The concentrations of pemetrexed and aumolertinib in H1975 and HCC827 tumor xenograft at 4 h post drug administration were assayed (*n* = 5). Con represents control; P represents pemetrexed; A represents aumolertinib; Osi represents osimertinib; P-A represents pemetrexed treatment followed by aumolertinib treatment. All of the data were expressed as the mean ± SEM, * *p* < 0.05 and *** *p* < 0.001

Next, we examined whether the tumoral vasculature normalization makes it more efficient for drug delivery. As shown in Fig. 6A, P-A sequence treatment did not change the plasma exposure of both pemetrexed and aumolertinib, indicated by the identical drug concentration curve against time and similar kinetic parameters with monotherapy groups (Table S2). However, the intratumoral concentration of pemetrexed after P-A sequence administration was remarkably higher than that in pemetrexed treatment group with 4.66-fold increase in H1975 tumors and 8.61-fold increase in HCC827 tumors (Fig. 6B). In contrast, the accumulation of pemetrexed in other major organs showed no significant difference between P-A sequence and pemetrexed treatment group (Fig. S5). Besides, P-A exhibited negligible influences on the accumulation of aumolertinib in the tumor mass.

### Superior anti-tumor effect of P-A sequence in patients harboring EGFR mutant

From April, 2020 to January, 2022, 50 patients had received aumolertinib monotherapy as first-line therapy and 15 patients received combination therapy (pemetrexed administered one week prior to aumolertinib). As shown in Table 1, no significance was observed in the age distribution, sex distribution and EGFR mutant type distribution in two groups. After two-cycle treatment, 14 patients (93.3%) in combination therapy group exhibited partial response revealed by more than 30% reduction in the longest diameter of target lesion. In comparison, only 64% patients exhibited partial response in aumolertinib monotherapy. Notably, no patients exhibited tumor progression after combination therapy group while 4 patients (8%) underwent target lesion increases by at least 20% or the appearance of new lesion after aumolertinib monotherapy. Overall, the objective response rate (ORR) and disease control rate (DCR) in combination therapy group are 93.3% and 100% respectively, obviously higher than 64% and 92% in aumolertinib monotherapy group.

**Table 1:** Characteristics of patients and therapeutic effect in the combined treatment group and the aumolertinib monotherapy group

	**Combined treatment (*n* = 15)**	**Aumolertinib (*n* = 50)**	***P*-value**
	**N (%)**	**N (%)**	
**Gender**			***p* = *0.25***
Male	9 (60%)	21 (42%)	
Female	6 (40%)	29 (58%)	
**Age**			***p* = *0.91***
Median Age	65	67	
< 70 years	9 (60%)	29 (58%)	
≥ 70 years	6 (40%)	21 (42%)	
**Stage prior therapy**			***p* = *0.77***
Stage II	0	2 (4%)	
Stage III	3 (20%)	10 (20%)	
Stage IV	12 (80%)	38 (76%)	
**EGFR**			***p* = *0.57***
Mutant	15 (100%)	50 (100%)	
Del19	9 (60%)	25 (50%)	
L858R	6 (40%)	25 (50%)	
**Tumor response**			***p* = *0.04***
Complete response (CR)	0 (0%)	0 (0%)	
Partial response (PR)	14 (93.3%)	32 (64%)	
Stable disease (SD)	1 (6.7%)	14 (28%)	
Progressive disease (PD)	0 (0%)	4 (8%)	

**Fig. 7:**
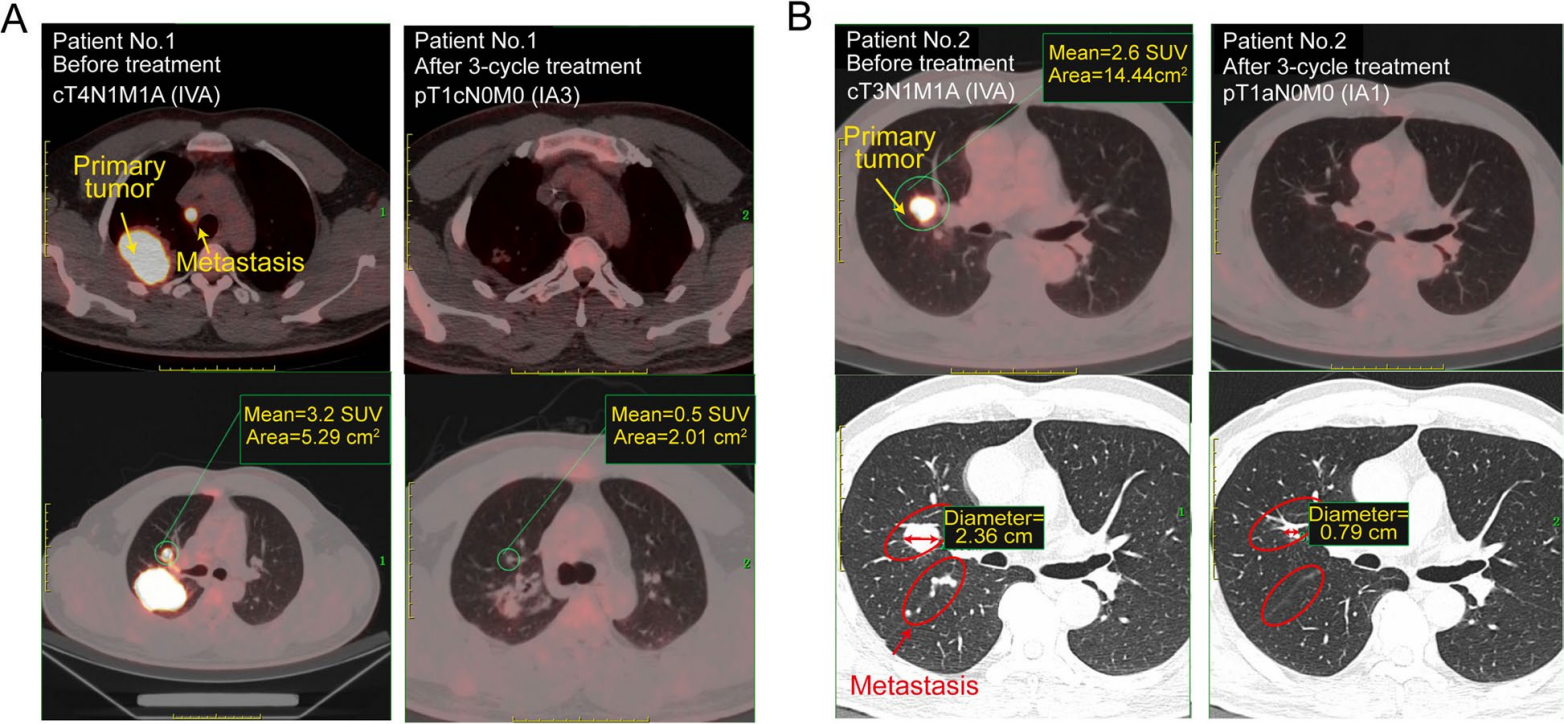
Positron emission tomography/Computed tomography (PET/CT) of representative NSCLC patients with metastasis before and after P-A combination therapy**.** **A** Patient No.1 harboring EGFR del19 mutant, TP53 mutation and EGFR amplification with pleural metastases. **B** Patient No.2 harboring EGFR del19 mutant and TP53 mutation with multiple pleural metastases. Arrows represent where the primary tumor and metastasis were performed

For five representative cases who received combination therapy (pemetrexed administered one week prior to aumolertinib) as neoadjuvant therapy, the significant tumor regressions were observed in patients harboring EGFR mutant after 2–3 cycle treatment (Table 2), independent of the different concomitant mutations. Pathological response was assessed by local pathologists, who measured the percentage of residual viable tumor in primary tumors resected from each patient during surgery. Surprisingly, four patients reached major pathological response changing from clinical stage III/IV to postoperative pathological stage I and one patient reached complete pathological response changing from clinical stage IIIB to postoperative pathological T0N0M0. Thereinto, two patients (Patient No.1 and No.2) occurred tumor metastasis and PET/CT was performed for further evaluation. As shown in Fig. 7, both the primary tumor and the metastatic lesion regressed significantly. Meanwhile, the priorly high accumulation of [18] F-FDG disappeared or significantly decreased.

**Table 2:** Five representative cases who were received combination therapy as neoadjuvant therapy

Patient No	No.1	No.2	No.3	No.4	No.5
**Age**	32	47	64	68	60
**Gender**	male	male	male	male	female
**Histology**	adenocarcinoma	adenocarcinoma	squamous carcinoma	adenocarcinoma	squamous carcinoma
**Clinical stage**	cT4N1M1 (IVA)	cT3N1M1 (IVA)	cT4N3M0 (IIIC)	cT3N2M0 (IIIB)	cT4N2M0 (IIIB)
**Molecular aberration**	EGFR 19del	EGFR 19del	EGFR 19del	EGFR L858R	EGFR 19del
**Concomitant mutations**	TP53 mutation, EGFR amplification	TP53 mutation	EGFR amplification	TP53 mutation, HEBB2 and EGFR amplification	-
**PD-L1 expression**	5%	0%	90%	0%	0%
**Neoadjuvant therapy cycles**	3	3	2	2	2
**Postoperative pathological stage**	pT1cN0M0 (IA3)	pT1aN0M0 (IA1)	pT1bN0M0 (IA2)	pT1bN0M0 (IA2)	pT0N0M0
**Postoperative pathological evaluation**	MPR	MPR	MPR	MPR	CPR

## Discussion

﻿For most EGFR-mutant NSCLC patients with third-generation EGFR TKIs treatment, resistance arises after a dramatic initial response to EGFR TKIs followed by stable minimal residual disease and subsequent development of drug-resistant tumors. The upfront third-generation EGFR TKIs-based combination therapy is promising to prevent and overcome the acquired resistance, however compelling clinical evidence indicated the failure of the combination of third-generation EGFR TKIs with either ICIs or antiangiogenic agents according to the negligible improvement on mOS and also the non-ignorable safety problems9,10. In comparison, combined treatment of third-generation EGFR TKIs and chemotherapy is more promising, which has a proven clinical tolerance17,18. Although several clinical researches have explored the interaction of first-generation EGFR-TKIs and cytotoxic agents in EGFR sensitive mutant patients, hitherto, only limited preclinical and clinical outcomes can be found for the combination of chemotherapy with third-generation EGFR TKIs.

Herein, we firstly performed a systemic assay in vitro to determine the combinatorial effect of pemetrexed and a novel marketed third-generation EGFR-TKI aumolertinib. During the experiment, we designed three combination strategies involving different administration orders and evaluated their integration effect on tumor cell growth with several NSCLC cell lines harboring distinct EGFR mutation. Surprisingly, administration orders of pemetrexed and aumolertinib played an unexpectedly decisive role on the final effects in cell lines harboring EGFR mutation (Fig. 1). Both cell survival assay and colony formation assay indicated that only P-A sequence exhibited synergic effect. In comparison, P + A just presented an addictive effect and A-P even exerted an obviously antagonistic effect. ﻿Different from EGFR-mutant cell H1975 and HCC827, all combination strategies exhibited antagonistic effect in EGFR-wide type cell A549. This distinction can be explained by that H1975 and HCC827 were much more sensitive to aumolertinib due to a high selectivity against EGFR mutation of aumolertinib, whereas A549 harboring wide type EGFR cannot exert comparable sensitivity to aumolertinib treatment (Fig. S1). Resembling to the various integration effects in vitro*,* three treatment sequence showed a significant difference on anti-tumor effect in vivo*,* among which, P-A displayed a superior anti-tumor effect over P + A and A-P (Fig. 3A-C). Furthermore, the synergic anti-tumor effect of P-A was further determined by the comparison with pemetrexed and aumolertinib monotherapy. We found that P-A presented an obvious tumor growth inhibition at the earliest and the most extent among all the drug treatment groups either in H1975 tumor bearing mice or HCC827 tumor bearing mice (Fig. 3 and Fig. S2).

High tumor metastasis rate is the hallmark of NSCLC. It is estimated that nearly two-thirds of NSCLC patients show evidence of local or distant metastasis involving the bones, brain and liver at the time of diagnosis. And only approximately 15% patients with metastatic NSCLC survive more than 5 years [31]. Despite the significant advancements in currently available therapies, the fairly high frequencies of tumor metastasis and recurrence posttreatment remain to be the most serious challenge in clinic [32]. We next asked whether the sequence-dependent synergistic effect appeared among the inhibition of cell migration and invasion. Consequently, P-A sequence treatment was one and only combined strategy showing the significantly higher inhibition of tumor cell migration than aumolertinib monotherapy in the wound healing assay and this synergistic effect was further proved by transwell migration and invasion assay as well as the inhibition of EMT-related factors (Fig. 2). Furthermore, P-A sequence also obviously decreased the possibility of liver metastasis in vivo (Fig. 3H and I).

Together with the in vitro and in vivo assays, we concluded a sequence-dependent synergistic effect of aumolertinib and pemetrexed on EGFR-mutant tumor growth and metastasis. Not limiting to the combination of aumolertinib and pemetrexed in our study, sequence-dependent difference was also observed in the preclinical researches involving the combination of chemotherapy with the first-generation EGFR TKI icotinib [33] or erlotinib [34], as well as the combination of ﻿the other third-generation EGFR TKI osimertinib with cisplatin [35], implying the ubiquitously important role of administration order among the EGFR TKIs-combination with chemotherapy. Although the synergism generated with the sequence of EGFR TKIs given following pemetrexed has gained attentions as a superior regimen, the underlying mechanisms are not well understood. Suppression of EGFR signaling pathway was the crucial mechanism for the anti-proliferation and anti-metastasis effect of EGFR TKIs. Despite no direct effect of pemetrexed on EGFR signaling pathway, the levels of p-AKT and p-ERK expression in P-A sequence treatment group were much lower than the aumolertinib (Fig. 4). While, when sequence alternating, A-P exhibited inferior suppression on the p-AKT and p-ERK expression in the comparison with aumolertinib monotherapy (Fig. S3). Similarly, the inferior suppression of ﻿p-AKT and p-ERK was also observed when treated with icotinib followed by pemetrexed (I-P), in accordance to its inferior effect [33]. These data suggested the synergistic effect of P-A depends on the enhanced inhibition of intracellular transduction pathways.

Intriguingly, our studies serendipitously found the relative abundant vessel nets surrounding H1975-induced xenograft and HCC827-induced xenograft by the comparison with A549-induced xenograft (data not shown). Meanwhile, a recent study uncovered the dominant regulation of EGFR on HIF-1α and VEGF in EGFR mutant NSCLC cells [29]. Accordingly, we anticipated the inhibition of EGFR signaling can influence the secretion of VEGF from the EGFR mutant tumor cells and consequently regulate intratumoral vessel growth. As inferred, we observed a significant reduction of VEGF secretion after aumolertinib treatment both in vitro and in vivo (Fig. 5A-C) and a normalization of pathological vascular angiogenesis (Fig. 5D-G). Herein, the newly found overlap of the vascular regulation between the EGFR-TKIs and antiangiogenic therapy could partially explain the negligible improvement of their clinical combination. Previously, it has been widely demonstrated that the intratumoral drug delivery could be obviously enhanced and subsequent better anti-tumor effect along with the tumor vessel normalization exerted by anti-angiogenesis agents [36]. Similarly, the concentration of pemetrexed after P-A sequence administration was remarkable higher than that in pemetrexed treatment group with no change of plasma exposure and accumulation in other major organs (Fig. 6**, **Fig. S5 and Table S2). The increased accumulation of pemetrexed in tumor facilitated a superior anti-tumor effect. Notably, to our knowledge, it is the first time to explain the synergic effect of EGFR TKIs and chemotherapy from the kinetic consideration.

At last, we sought to investigate whether the resultant conclusion derived from pre-clinical models in our study is meaningful to be applied to the clinic. Due to the lack of clinical trials involving third-generation EGFR TKIs and pemetrexed, we firstly compared the completed clinical trials about the combination regimen of first-generation EGFR TKIs and chemotherapy (Table S3). Three independent clinical trials indicated that sequential administration of erlotinib or gefitinib following chemotherapy led to a statistically significant improvement in PFS and mOS than chemotherapy alone [37–39], while concurrent either erlotinib or gefitinib with chemotherapy failed to confer a survival advantage over chemotherapy alone [40–42]. Meanwhile, the ﻿combination of chemotherapy and gefitinib also provided a better survival benefit than gefitinib monotherapy for patients with lung adenocarcinoma harboring EGFR mutations by first using cytotoxic drug on day 1 and gefitinib from day 5–21 [39]. While, when the sequential alternating, gefitinib given before chemotherapy exhibited a significantly worse outcome [43]. Consequently, in accordance with the preclinical trails, administration sequence in the TKI-based combination with chemotherapy indeed plays a decisive role on the final outcomes in clinic. In 2021, an open-label randomized phase 2 study indicated the addition of chemotherapy to osimertinib as a second-line treatment did not prolong survival, though it was found to be generally tolerable. Based on our study and the above clinical reports of first-generation EGFR TKIs, we suggested that the inappropriate regimen design was the main cause for the recently neglectable improvement of osimertinib-cytotoxic chemotherapy combined treatment, in which combination group received concurrent treatment of osimertinib and carboplatin/pemetrexed in a 3-week cycle for up to four cycles [44], a schedule (P + A) that in our study had shown only addictive effect in vitro and mediocre anti-tumor effect in vivo (Fig. 1,2,3). In comparison, we proposed that pemetrexed administered prior to third-generation EGFR TKIs would be a favorable choice for clinical. ﻿Indeed, a clinical retrospective study proved it. From April, 2020 to January, 2022, 15 patients priorly received pemetrexed/cisplatin and following aumolertinib in 21-day cycle. As shown in Table 1, patients in this sequence combination therapy group exhibited significantly superior tumor response than aumolertinib monotherapy. Five representative cases further revealed the satisfactory therapeutic effect of P-A sequence treatment as neoadjuvant therapy, consequently indicating the promising application of P-A sequence treatment in clinic (Table 2 and Fig. 7). Despite current clinical retrospective study proved the superiority of the regimen that pemetrexed was administered prior to third-generation EGFR TKIs, further systemic clinical trials are indispensable to determine whether administration sequence play a crucial role on the clinical outcomes during receiving aumolertinib and pemetrexed.

## Conclusion

Herein, our study proved that administration order of pemetrexed and aumolertinib played a decisive role in the final therapeutic effects. Preclinical assay revealed that only aumolertinib treatment following pemetrexed (P-A) exhibited synergistic inhibition on tumor growth and metastasis, by the enhanced suppression of EGFR pathway as well as increased intratumoral accumulation of pemetrexed through aumolertinib-mediated reduction of VEGF secretion and consequently tumor vessel normalization. Notably, this conclusion derived from pre-clinical models is also meaningful for the clinic, proved by the superior effect of P-A in the clinical retrospective study. Collectively, we highlight the importance of administration order of chemotherapy and aumolertinib in combination therapy and offer a promising combination schedule for NSCLC patients harboring EGFR mutant, providing a strong support to future clinical regimen design.

## Supplementary Information

**Additional file 1: Figure S1. **The IC_50_ of pemetrexed andaumolertinib on different NSCLC cell lines. A549,HCC827 and H1975 were exposure to a series concentration of pemetrexed** A **and aumolertinib** B**, and cell survival rates were measured at 72 hours post-administration(*n*=6). IC_50_ values were calculated by fitting the dose-responsecurves with equation “Y=Bottom+(Top-Bottom)/(1+(IC_50_/X)^Hillslope)”. All of the data were expressed as the mean ± SEM. **FigureS2. **The daily change of xenograft volume and mice weight in H1975 and HCC827bearing mice.** A **Increase folds of tumor volumes at different days post drug administrationrelated to the initial volumes were shown. * *p*<0.05,** *p*<0.01, *** *p*<0.001versus the control groups and ^#^*p*<0.05,^##^*p*<0.01, ^###^p<0.001 versus the P-A sequence treatment.** B **Miceweights were monitored during drug treatment. All of the data were expressed asthe mean ± SEM.** Figure S3. **EGFR pathway in H1975 tumor mass after differentdrug administration. **FigureS4. **Awestern blot assay was performed to detect the expression of the representativeproteins within EGFR pathway in H1975 cell line post aumolertinib (2 μM) and osimertinib(2 μM) treatment for 24 hours.** Figure S5. **Concentrations of aumolertinib and pemetrexed inmain tissues. **Table S1.** Detailed information of primers used inthis study. **Table S2. **Pharmacokineticsparameters in different groups were compared. **Table S3. **Completedclinical trials involving the combination of EGFR TKIs and chemotherapy inpatients with NSCLC.

## Data Availability

The datasets used and analyzed in this study are available from the corresponding author upon reasonable request.

## Abbreviations

A: Aumolertinib
P: Pemetrexed
P + A: Concomitant treatment with pemetrexed and aumolertinib
P-A: Pemetrexed treatment followed by aumolertinib treatment
A-P: Aumolertinib treatment followed by pemetrexed treatment
Osi: Osimertinib
EGFR: Epidermal Growth Factor Receptor
EMT: Epithelial-Mesenchymal Transition
HUVEC: Human Umbilical Vein Endothelial Cell
ICIs: Immune Checkpoint Inhibitors
Mos: Median Overall Survival
NSCLC: Non-Small Cell Lung Cancer
PFS: Progression-Free Survival
PET/CT: Positron Emission Tomography/Computed Tomography
TKI: Tyrosine Kinase Inhibitor
VEGF: Vascular Endothelial Growth Factor
