# Supplementary material for: Sequence-dependent synergistic effect of aumolertinib-pemetrexed combined therapy on EGFR-mutant non-small-cell lung carcinoma with pre-clinical and clinical evidence
Source: J Exp Clin Cancer Res. 2022 May 3;41:163. doi: 10.1186/s13046-022-02369-3 (PMC9063085; doi:10.1186/s13046-022-02369-3)
Supplement: Supplementary file 1 — Additional file 1: Figure S1. The IC50 of pemetrexed andaumolertinib on different NSCLC cell lines. A549,HCC827 and H1975 were exposure to a series concentration of pemetrexed A and aumolertinib B, and cell survival rates were measured at 72 hours post-administration(n=6). IC50 values were calculated by fitting the dose-responsecurves with equation “Y=Bottom+(Top-Bottom)/(1+(IC50/X)^Hillslope)”. All of the data were expressed as the mean ± SEM. FigureS2. The daily change of xenograft volume and mice weight in H1975 and HCC827bearing mice. A Increase folds of tumor volumes at different days post drug administrationrelated to the initial volumes were shown. * p<0.05,** p<0.01, *** p<0.001versus the control groups and #p<0.05,##p<0.01, ###p<0.001 versus the P-A sequence treatment. B Miceweights were monitored during drug treatment. All of the data were expressed asthe mean ± SEM. Figure S3. EGFR pathway in H1975 tumor mass after differentdrug administration. FigureS4. Awestern blot assay was performed to detect the expression of the representativeproteins within EGFR pathway in H1975 cell line post aumolertinib (2 μM) and osimertinib(2 μM) treatment for 24 hours. Figure S5. Concentrations of aumolertinib and pemetrexed inmain tissues. Table S1. Detailed information of primers used inthis study. Table S2. Pharmacokineticsparameters in different groups were compared. Table S3. Completedclinical trials involving the combination of EGFR TKIs and chemotherapy inpatients with NSCLC. [file 13046_2022_2369_MOESM1_ESM.docx]

**Supporting Information**


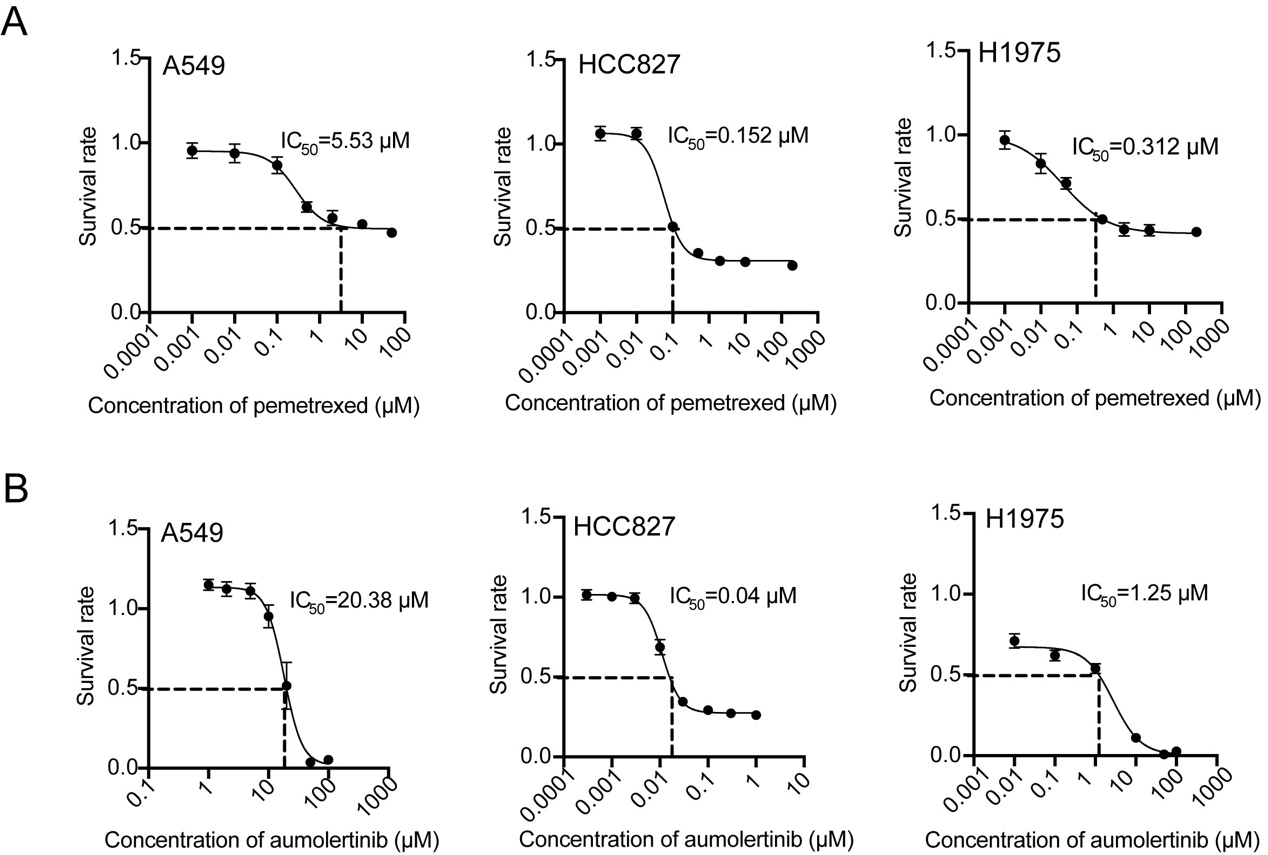


**Figure S1. The IC_50_ of pemetrexed and aumolertinib on different NSCLC cell lines.** A549, HCC827 and H1975 were exposure to a series concentration of pemetrexed (A) and aumolertinib (B), and cell survival rates were measured at 72 hours post-administration (n=6). IC_50_ values were calculated by fitting the dose-response curves with equation “Y=Bottom+(Top-Bottom)/(1+(IC_50_/X) ^Hillslope)”. All of the data were expressed as the mean ± SEM.


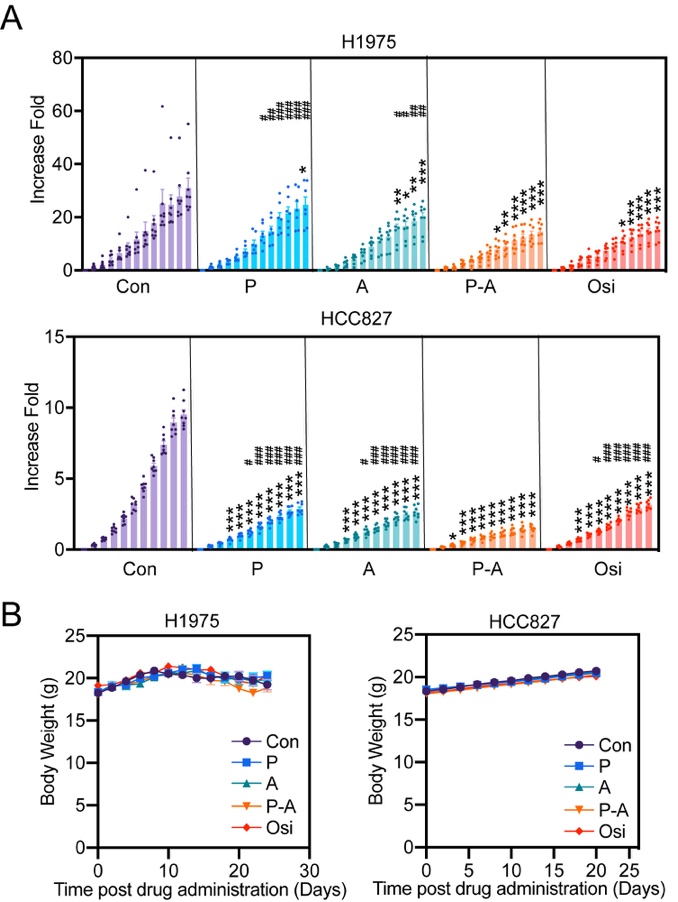


**Figure S2. The daily change of xenograft volume and mice weight in H1975 and HCC827 bearing mice.** (A) Increase folds of tumor volumes at different days post drug administration related to the initial volumes were shown. * p<0.05, ** p<0.01, *** p<0.001 versus the control groups and ^#^ p<0.05, ^##^ p<0.01, ^###^ p<0.001 versus the P-A sequence treatment. (B) Mice weights were monitored during drug treatment. All of the data were expressed as the mean ± SEM.


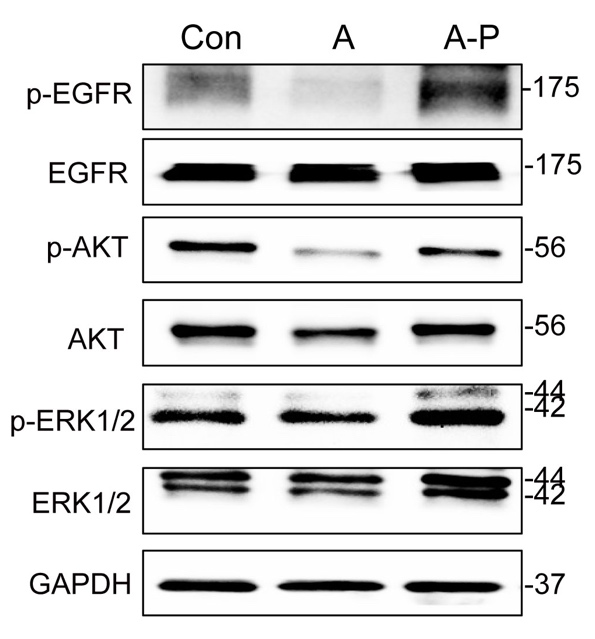


**Figure S3. EGFR pathway in H1975 tumor mass after different drug administration.**


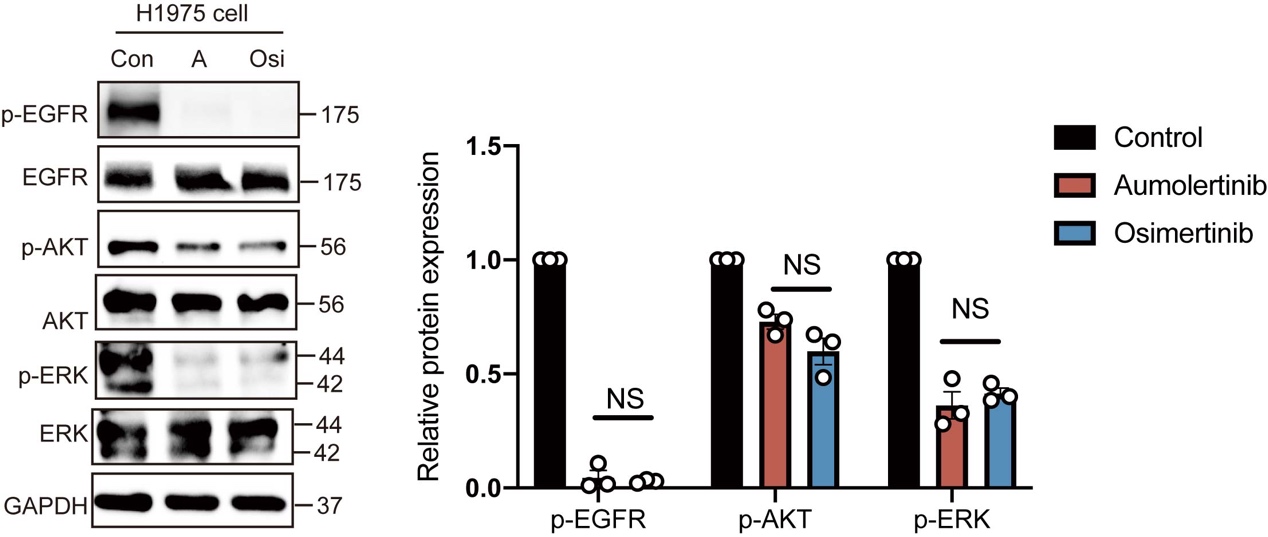


**Figure S4. A western blot assay was performed to detect the expression of the representative proteins within EGFR pathway in H1975 cell line post aumolertinib (2 μM) and osimertinib (2 μM) treatment for 24 hours.**


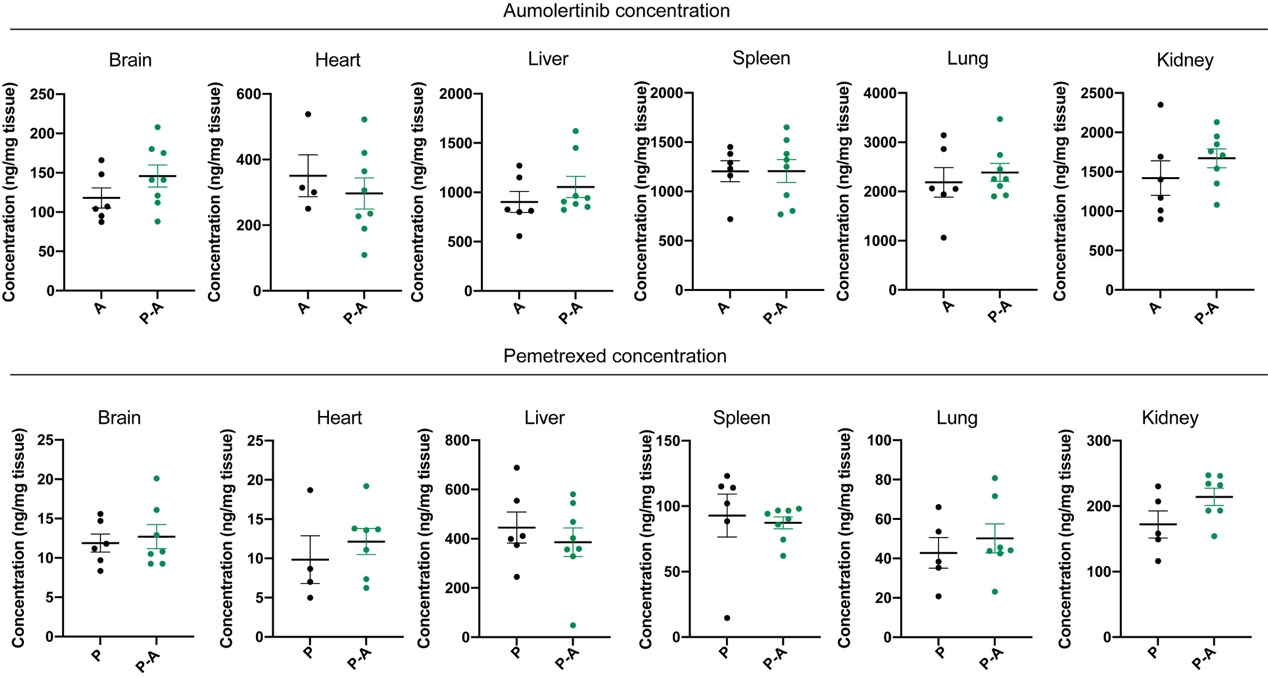


**Figure S5. Concentrations of aumolertinib and pemetrexed in main tissues.**

**Table.S1 Detailed information of primers used in this study.**

| **Gene** | **Species** | **Primer Forward（5'→3’）** | **Primer Reverse（3'→5’）** |
| --- | --- | --- | --- |
| β-actin | Human | GCGTGACATTAAGGAGAAG | GAAGGAAGGCTGGAAGAG |
| HIF-1α | Human | GGTTCTCACAGCTGATGGTG | TTCTTCCTCGGCTAGTTAGG |
| VEGF | Human | CGACGGCTTGGGGAGATTGC | GGGCGGTGTCTGTCTGTCTG |
| TGF-β | Human | TTCGCCTTAGCGCCCACTGC | GGCCGGTAGTGAACCCGTTG |
| ANG | Human | GCACGAAGACCAACAACAAA | CTTCTTTCCATTGTCCTGCC |
| SFLT | Human | TGGCCTTCCCTGACTTCAGGTGTG | GTTCCTTGTAGCCAGTCTTCACC |
| Angiostatin | Human | AGGAACAGCTGCACAAAGGA | AGTCAAGCAGTCGGTGACAG |

**Table.S2 Pharmacokinetics parameters in different groups were compared.**

| **Mice**  **Models** | **Tested**  **Drug** | **Groups** | **T_1/2_**  **(h）** | **T_max_**  **(h)** | **C_max_**  **(ng/mL)** | **AUC_0-t_**  **(μg/L·h)** | **AUC_0-∞_（μg/L·h）** | **Cl**  **(L/h·kg)** |
| --- | --- | --- | --- | --- | --- | --- | --- | --- |
| HCC827 Xenografts | Aumolertinib | Aumolertinib | 8.05 | 1 | 1750.0 | 15357.4 | 20732.9 | 242.4 |
|  |  | P-A | 8.14 | 1 | 1814.0 | 15559.4 | 20738.5 | 241.3 |
|  | Pemetrexed | Pemetrexed | 0.52 | 0.25 | 62933.3 | 35868.1 | 36157.2 | 1373.3 |
|  |  | P-A | 0.60 | 0.25 | 58760.0 | 35239.0 | 35420.2 | 2774.5 |
| H1975 Xenografts | Aumolertinib | Aumolertinib | 4.09 | 2 | 2670.0 | 24574.8 | 25005.6 | 2824.0 |
|  |  | P-A | 3.31 | 2 | 2277.5 | 18578.6 | 18703.9 | 1069.3 |
|  | Pemetrexed | Pemetrexed | 0.44 | 0.25 | 123333.3 | 72619.1 | 72817.6 | 1373.3 |
|  |  | P-A | 0.45 | 0.25 | 88800.0 | 56150.2 | 56322.4 | 1775.3 |

**Table.S3 Completed clinical trials involving the combination of EGFR TKIs and chemotherapy in patients with NSCLC.**

| **Author** | **Regimen** | **Dose** | **Outcome** |
| --- | --- | --- | --- |
| Gatzemeier, U. *et al^39^* | Combination: gemcitabine on day 1 and 8 plus cisplatin on day 1 with erlotinib on day1-21  Monotherapy: gemcitabine on day 1 and 8 plus cisplatin on day 1 with placebo on day1-21 | Erlotinib:150 mg/d, *orally*  Placebo: 150 mg/d, *orally*  Gemcitabine:1,250mg/m^2^  Cisplatin: 80 mg/m^2^, *iv* | Combination ≈  Chemotherapy monotherapy |
| Herbst, R. S. *et al ^41^* | Combination: paclitaxel on day 1 plus carboplatin on day 1 with erlotinib on day1-21  Monotherapy: paclitaxel on day 1 plus carboplatin on day 1 with placebo on day1-21 | erlotinib:150 mg/d, *orally*  placebo: 150 mg/d, *orally*  carboplatin: 5 AUC *iv*  paclitaxel :200mg/m^2^, *iv* | Combination ≈  Chemotherapy monotherapy |
| Herbst, R. S. *et al ^40^* | High dose combination: paclitaxel on day 1 plus carboplatin on day 1 with Gefitinib (500 mg/d) on day 1-21  Low dose combination: paclitaxel on day 1 plus carboplatin on day 1 with Gefitinib (250 mg/d) on day 1-21  Monotherapy: paclitaxel on day 1 plus carboplatin on day 1 with placebo on day1-21 | gefitinib:500mg/d, *orally*  gefitinib:250mg/d, *orally*  placebo: 150 mg/d, *orally*  carboplatin: 5 AUC *iv*  paclitaxel :225mg/m^2^, *iv* | High and low dose Combination ≈  Chemotherapy monotherapy |
| Wu, Y. L. *et al.^36^* | Combination: Gemcitabine on day 1 and 8 plus Platinum (carboplatin or cisplatin) on day1 with erlotinib on day 15–28  Monotherapy: Gemcitabine on day 1 and 8 plus Platinum (carboplatin or cisplatin) on day1 with placebo on day 15–28 | gemcitabine:1250 mg/m², *iv*  carboplatin :5 AUC, *iv*  cisplatin: 75 mg/m², *iv*  erlotinib :150 mg/d, *orally* | Combination>  Chemotherapy monotherapy. |
| Mok, T. S. *et al.^37^* | Combination: Erlotinib on days 15 to 28 of a 4-week cycle that included gemcitabine on days 1 and 8 and either cisplatin on day1 or carboplatin on day1  Monotherapy: Placebo on days 15 to 28 of a 4-week cycle that included gemcitabine on days 1 and 8 and either cisplatin on day1 or carboplatin on day1 | erlotinib:150mg/d, *orally*  placebo: 150 mg/d, *orally*  gemcitabine:1,250mg/m^2^, *iv*  cisplatin:75 mg/m^2^, *iv*  carboplatin :5 AUC, *iv* | Combination>  Chemotherapy monotherapy. |
| Han, B. *et al. ^38^* | Combination: gefitinib on Days 5–21 combined with pemetrexed on Day 1 and carboplatin on Day 1 repeated every four weeks for up to six cycles  Chemotherapy group: the same regimen; only pemetrexed every four weeks  Gefitinib group: the same regimen; only gefitinib every four weeks | pemetrexed:500 mg/m^2^, *iv*  carboplatin: 5AUC, *iv*  gefitinib :250 mg/d, *orally* | Combination therapy＞Chemotherapy  Combination therapy＞Gefitinib |
| Oizumi, S. *et al. ^42^* | Concurrent combination: gefitinib daily + carboplatin /pemetrexed on day1 in a 3-week cycle up to six cycles, followed by gefitinib +pemetrexed maintenance  TKI pretreatment combination: gefitinib daily for 8 weeks, followed with two cycles of carboplatin/pemetrexed, then maintained by alternating gefitinib and pemetrexed | gefitinib: 250 mg/d, *orally*  carboplatin:6 AUC, *iv*  pemetrexed:500mg/m^2^, *iv* | Concurrent combination＞  TKI pretreatment combination |
| Tanaka, K. *et al ^43^* | Combination: the combination of osimertinib with carboplatin-pemetrexed (carboplatin on day 1 and pemetrexed on day 1 in a 3-week cycle for up to four cycles)  Monotherapy：osimertinib alone | osimertinib:80 mg/d, *orally*  carboplatin: 5 AUC, *iv*  pemetrexed:500 mg/m^2^, *iv* | Combination ≈  Osimertinib monotherapy |
